# The Involvement of the Peptidergic Systems in Breast Cancer Development

**DOI:** 10.3390/cancers17223662

**Published:** 2025-11-14

**Authors:** Manuel L. Sánchez, Prema Robinson, Zal Italia, Tan Hoang, Miguel Muñoz, Rafael Coveñas

**Affiliations:** 1 Laboratory of Neuroanatomy of the Peptidergic Systems, Institute of Neurosciences of Castilla and León (INCYL), University of Salamanca, 37007 Salamanca, Spain; lisardosanchez8@gmail.com; 2 Department of Infectious Diseases, Infection Control and Employee Health, The University of Texas MD Anderson Cancer Center, Houston, TX 77030, USA; probinson1@mdanderson.org (P.R.); zsitalia1@mdanderson.org (Z.I.); tmhoang@mdanderson.org (T.H.); 3 Research Laboratory on Neuropeptides (IBIS), Virgen del Rocío University Hospital, 41013 Sevilla, Spain; miguel.mmunoz@gmail.com; 4 Group GIR USAL: BMD (Bases Moleculares del Desarrollo), University of Salamanca, 37007 Salamanca, Spain

**Keywords:** bradykinin, corticotropin-releasing factor, glucagon-like peptide 1, kisspeptin, neuromedin, neuropeptide Y, substance P, oncogenic peptides, anticancer peptides, peptide receptor antagonists

## Abstract

The current known data on the involvement of the peptidergic systems in breast cancer progression is overwhelmingly vast. Peptidergic systems are useful tools for imaging, diagnosis, prognosis and treatment of breast cancer. These systems play a crucial role in both basic and clinical breast cancer research by enabling the exploration of novel molecular mechanisms, signaling pathways, and the development of effective drug design strategies. Breast cancer cells overexpress peptide receptors; at the same time they are known to interact with peptides that (a) exert an oncogenic action (adrenomedullin 2, endothelin, gastrin-releasing peptide, neurokinin A, neuromedin, neuropeptide Y, neurotensin, substance P, vasoactive intestinal peptide), (b) exert an anticancer action (angiotensin (1–7), ghrelin, peptide YY) or (c) exert dual oncogenic and anticancer effects (adrenomedullin, angiotensin II, bradykinin, corticotropin-releasing factor, β-endorphin, glucagon-like peptide 1, gonadotropin-releasing hormone, kisspeptin, methionine-enkephalin, oxytocin). This indicates that peptides, as well as peptide receptor agonists and antagonists, may serve as antitumor agents due to their diverse actions against breast cancer development, including the inhibition of cell proliferation, migration and invasion, induction of apoptosis, and anti-angiogenesis. Multiple strategies have been developed to combat breast cancer, including peptide receptor silencing; antibodies conjugated to specific signaling proteins; antibodies targeting specific peptide receptors or oncogenic peptides; and the use of peptides or peptide receptor agonists/antagonists loaded with antitumor cargo. Future lines of research are suggested in breast cancer using promising anti-breast-cancer peptide receptor antagonists (HOE-140, exendin (9–39), bosentan, macitentan, PD168,368, CGP71,683A, SR48,692, aprepitant) or agonists (FR190,997, semaglutide, exendin 4, goserelin) mentioned in this review. Peptidergic systems have tremendous anti-breast-cancer clinical potential which must be exploited and developed. Taken together, the available data highlight the enormous promise of translational research into breast cancer and peptidergic systems for the development of effective treatments. A full understanding of the roles played by the peptidergic systems in breast cancer will serve to improve diagnosis and treatment.

## Simple Summary

Breast cancer cells overexpress peptide receptors and interact with peptides that (a) exert an oncogenic action, (b) exert an anticancer action or (c) exert dual oncogenic and anticancer effects. This indicates that peptides, as well as peptide receptor agonists and antagonists, may serve as antitumor agents due to their diverse actions against breast cancer development, including the inhibition of cell proliferation, migration, and invasion, the induction of apoptosis, and anti-angiogenic effects. Peptidergic systems have great anti-breast-cancer clinical potential which must be exploited and developed. A greater understanding of the roles played by the peptidergic systems in breast cancer will serve to improve diagnosis and treatment.

## 1. Introduction

By 2040, an estimated 28.4 million people worldwide are expected to be diagnosed with cancer [1]. Although progress in the diagnosis and treatment of this disease has advanced immensely in recent years, new lines of research along with the development of novel therapeutic strategies are necessary to combat this deadly disease. More specifically, (a) novel molecular targets to counteract cancer development and progression as well as (b) novel compounds that destroy tumor cells without effects on normal cells of the body are needed in current cancer research. One of the promising lines of research is peptides and their receptors. Bioactive peptides, via peptide receptors (G protein-coupled receptors), play important roles in cell communication, proliferation, migration, survival and mitogenesis and are involved in numerous physiological actions and pathologies, including cancer [2]. The causes of malfunction of G protein-coupled receptors are diverse, and malfunctions lead to overexpression, protein mutations, overstimulation (as a result of ligand overexpression), truncation, aberrant dimerization/oligomerization, and distorted internalization [3]. Thus, when peptide receptors fail to adequately regulate cellular functions (such as proliferation, migration, apoptosis, mitochondrial function, oxidative stress), tumors can manifest, further highlighting the direct relation between cancer and malfunction of peptide receptors. The blockade, dysfunction, or excessive stimulation of peptide receptors can promote cellular disturbances that contribute to tumor formation by altering cancer cell metabolism, enhancing proliferation and migration, inhibiting apoptosis, and increasing angiogenesis. This means that peptide receptors play a crucial role in cancer research, as they enable the development of tailored pharmacological strategies to block signaling pathways that promote cellular disturbances [3].

Numerous in vitro and in vivo studies have demonstrated that the peptidergic systems (bioactive peptides and their receptors) are involved in the development (tumor cell proliferation, migration and invasion, anti-apoptotic effect, angiogenesis) of many different types of cancer [4,5,6]. In this context, two crucial facts stand out: tumor cells overexpress peptide receptors compared to normal cells [7,8,9], and the survival of cancer cells is mediated by oncogenic peptide receptors (e.g., the neurokinin 1 receptor). Thus, it appears that tumor cells become hostages to the signals mediated by oncogenic peptides, overexpressing oncogenic peptide receptors to guarantee the reception of these signals. This is because the sources of oncogenic peptides are diverse and abundant: tumor cells themselves and cells within the tumor microenvironment synthesize and release peptides; peptides are also released from nerve terminals, and importantly, peptides can reach the tumor through the bloodstream [10]. Previous key findings open the door to new and promising anticancer strategies, as peptides (upon binding to their specific receptors) can exert either oncogenic or anticancer effects. This implies that peptide receptor antagonists, which promote apoptosis in cancer cells and inhibit cell migration and angiogenesis, as well as peptides or peptide analogs that deliver antitumor agents into cancer cells or directly exert anticancer effects, hold potential as effective antitumor therapies. Currently there is no bioactive peptide receptor antagonist approved as an antitumor agent by the United States Food and Drug Administration (FDA), but the FDA has approved the use of peptide analogs (e.g., gonadotropin-releasing hormone, somatostatin) for the diagnosis and treatment of some tumors [11]. The gonadotropin-releasing hormone analog named goserelin (Zoladex) has been approved in the palliative treatment of advanced breast cancer (BC) in both post- and pre-menopausal women [12]. Importantly, various types of cancer express the same peptide receptor, which means that if this receptor mediates an oncogenic response, a single anticancer treatment using the corresponding peptide receptor antagonist alone or in combination therapy with radiotherapy, chemotherapy or immunotherapy could be broadly applied.

BC is the leading cause of cancer mortality and the most diagnosed cancer in women. A thorough understanding of the role of the peptidergic systems in BC will serve to improve imaging, diagnosis, prognosis and treatment. The numerous peptidergic systems which are involved in the development of BC and possibly could offer new promising options for treatment of BC are reviewed and reported in this review. Furthermore, peptidergic system antagonists (e.g., neurokinin receptor 1 antagonist aprepitant, a morpholine derivative) in conjunction with chemotherapeutic drugs such as cisplatin or doxorubicin augmented the anticancer action against triple-negative BC cells and at the same time attenuated the harmful effects mediated by chemotherapy [13,14]. This review will highlight the tremendous BC clinical potential of the peptidergic system and highlight the importance for this system to be further developed and exploited as a therapeutic strategy for BC.

## 2. Breast Cancer and Peptidergic Systems

In the following sections we will individually review the following peptides involved in BC development: (a) oncogenic and anticancer peptides (adrenomedullin, angiotensin II, bradykinin, corticotropin-releasing factor, β-endorphin, enkephalin, glucagon-like peptide 1, gonadotropin-releasing hormone/luteinizing hormone-releasing hormone, kisspeptin, oxytocin); (b) oncogenic peptides (adrenomedullin 2, endothelin, gastrin-releasing peptide, neurokinin A, neuromedin, neuropeptide Y, neurotensin, substance P, vasoactive intestinal peptide); (c) anticancer peptides (angiotensin (1–7), ghrelin, peptide YY); and (d) other bioactive and non-bioactive peptides (ASRPS, carnosine, cocaine- and amphetamine-regulated transcript, dynorphin, galanin, HMK, KLA, LINC00511-133 aa, and melittin).

### 2.1. Oncogenic and Anticancer Peptides

#### 2.1.1. Adrenomedullin

Adrenomedullin (AM) is a multifunctional peptide hormone first discovered in human pheochromocytoma tissue in 1993 [15]. It plays important roles in cardiovascular homeostasis, angiogenesis, inflammation, and tumor biology [15]. AM binds to calcitonin receptor-like receptor when associated with receptor activity-modifying proteins (RAMP2 or RAMP3) [15]. Signaling primarily involves the cyclic adenosine monophosphate (cAMP)-protein kinase A (PKA) pathway and activates phosphoinositide 3-kinase (PI3K)/Akt and mitogen-activated protein kinase (MAPK)/extracellular signal-regulated kinase (ERK) pathways [16]. Overexpression of AM has been observed in various cancers; the oncogenic effects of adrenomedullin (AM) and AM2 (intermedin) in numerous types of cancer, including breast, lung, pancreatic, and prostate cancer and gliomas, have been widely reported [6,16]. AM antagonists (e.g., anti-AM antibodies, receptor blockers) are under investigation as anticancer therapies [16]. AM is upregulated in hypoxic tumor environments, thereby aiding adaptation of tumor cells to adverse environments. AM suppresses immune responses against tumors [16] and it is involved in cell proliferation and angiogenesis [15,17]. Tumor-expressed AM accelerated BC bone metastasis [18]; fibroblasts located in breast carcinomas favored angiogenesis and tumor growth via the release of AM [18,19]. However, a study has demonstrated that AM blocked BC cell invasion and metastasis; its expression was decreased in triple-negative BC cells and samples, and low expression was associated with an augmented risk of metastasis and recurrence and poor prognosis [17,18]. Furthermore, it has been shown that the release of AM from BC cells promoted lipolysis and browning of adipocytes [20]; this is important because (a) adipocytes supply lipids, which are used as an energy source by tumor cells, and (b) adipokines favor tumor progression [20,21]. Thus, the use of AM antagonists is a good potential target in BC but it seems that this strategy would not be useful for the treatment of triple-negative BC cells.

#### 2.1.2. Angiotensin II

Angiotensin II can promote tumor development and progression through several mechanisms, primarily via angiotensin II receptor 1 signaling [22]. Some of the tumorigenic features of angiotensin II are listed as follows: (a) angiotensin II stimulates cell proliferation through the MAPK/ERK and PI3K/Akt pathways; (b) angiotensin II promotes epithelial–mesenchymal transition (EMT), enhancing invasiveness; (c) angiotensin II upregulates VEGF (vascular endothelial growth factor) and angiopoietins, facilitating tumor vascularization; (d) angiotensin II stimulates fibroblast activation, matrix remodeling, and immune cell recruitment; and (e) it enhances expression of matrix metalloproteinases (MMPs), degrading extracellular matrix to allow invasion [23,24,25,26,27]. More specifically, angiotensin II is involved in BC development [9]. This peptide regulates BC cell proliferation and migration as well as angiogenesis, and the overexpression of the angiotensin II receptor 1 favors angiogenesis and BC cell growth [9]. This overexpression serves for the diagnosis and treatment of BC using, for example, ^68^Ga/^177^Lu-labeled angiotensin II [9]. Contrary to the above studies showing angiotensin II to regulate BC cell proliferation and migration, a study reported that, although angiotensin II decreased the motility of BC cells, no effect was observed regarding invasion and proliferation [28]. Angiotensin II favored macrophage polarization toward anti-inflammatory M2-like macrophages and pro-inflammatory M1-like macrophages [29]. Angiotensin II inhibited tumor growth/progression in MDA-MB-23 BC cells but promoted MCF-7 BC cell growth/progression [29]. Most studies showed that angiotensin II predominantly promotes tumor development and progression, and hence angiotensin II could possibly be harnessed for diagnosis and treatment of BC.

#### 2.1.3. Bradykinin

Bradykinin has been implicated in tumor progression, particularly by promoting angiogenesis and increasing vascular permeability [30]. Its receptors, especially B1, are upregulated in cancer, infection, and injury, making them potential drug targets. B2 receptor antagonists (e.g., icatibant) are used in treating hereditary angioedema [31]. Bradykinin receptor blockers are being explored for treating inflammation, neuropathic pain, and cancer [32]. Bradykinin activates bradykinin receptors, mainly B2 receptors [33]. B2 receptor stimulation triggers downstream signaling (e.g., MAPK/ERK and PI3K/Akt pathways), promoting cell proliferation, survival under stress and resistance to apoptosis [33]. Bradykinin increases MMP expression, especially MMP-9, which facilitates extracellular matrix degradation, a key step in cancer invasion and metastasis [34,35]. Through B2 receptors, bradykinin induces VEGF expression, promoting angiogenesis [36]. Bradykinin recruits immune cells and enhances cytokine and chemokine release, contributing to a pro-tumor inflammatory microenvironment [33,37].

More specifically with respect to BC, the kinin B2 receptor is overexpressed in some BC cells [38]. B2 receptor activates the oncogenic ERK pathway [39] and bradykinin promotes EMT, enabling BC cells to migrate and invade. Bradykinin facilitates the migration and invasion of BC cells (MDA-MB-231, MCF-7, T47D); these effects were blocked using kinin B1 and B2 receptor antagonists (Des-[Arg9]-Leu8-bradykinin, HOE-140) [38]. These antagonists also blocked tumor growth in in vivo experiments [38]. However, kinin receptor B1 and B2 agonists also exerted antiproliferative effects: kinin receptor B2 agonist FR190,997 had antiproliferative actions against MDA-MB-231/MCF-7 BC cells [39]. Cell-penetrant kinin receptor B1 antagonists exerted an anticancer effect against triple-negative BC cells: the toxic effect of these antagonists against MDA-MB-231 cells was higher than that observed against cells with low/non-expressing kinin receptor B1 [40]. Moreover, the authors reported that the kinin receptor B1 antagonist R934, which is unable to cross cell membranes, (a) did not exert an antitumor action against BC cells, and (b) kinin receptor B1 antagonists can cooperate with chemotherapeutic drugs (paclitaxel, doxorubicin) to facilitate the death of triple-negative BC cells [40]. The migration and invasion of tumor cells promoted by bradykinin was also inhibited when Src and FAK (focal adhesion kinase) inhibitors were administered [38]. These results highlight how bradykinin, through kinin B1 and B2 receptors, activates the migration and invasion of BC cells via the FAK/Src signaling pathways. Bradykinin analogs promoted the proliferation of BC cells and the release of MMP 2/9 from both estrogen-sensitive and -insensitive BC cells favoring invasion and metastasis [41,42]. The stimulation of estrogen-sensitive BC cells with kinin B1 receptor agonists increased the levels of peptidases kallikrein (KLK)11 and KLK6 (favoring invasiveness and proliferation) and decreased the level of KLK10, a protease related with growth suppression [41]. These agonists also promoted the release of KLK1 and KLK6, which is important for cell invasion and kinin production mechanisms [41]. Accordingly, kinin B1 and B2 receptor antagonists could serve as a treatment option for BC.

#### 2.1.4. Corticotropin-Releasing Factor

Corticotropin-releasing factor (CRF), also known as corticotropin-releasing hormone, is a hypothalamic peptide that plays a central role in the stress response by activating the hypothalamic–pituitary–adrenal axis [43]. However, beyond its endocrine role, CRF and its receptors (CRF 1 and CRF 2) are involved in various extrahypothalamic functions, including inflammation, immune regulation, and importantly, cancer biology [44]. CRF signaling can promote tumor cell proliferation, inhibit apoptosis, and enhance survival [44,45]. This is particularly relevant in cancers where CRF or CRF receptors are overexpressed, such as colorectal, breast, and prostate cancers [46,47]. CRF may contribute to angiogenesis, facilitating tumor nourishment and growth [48]. CRF can modulate the tumor microenvironment (TME) and promote invasion and metastasis by activating MMPs and inducing EMT [49,50].

CRF receptors have been demonstrated in BC samples and cells [51,52,53,54,55]. BC samples and benign adjacent tissues express CRF 1 and 2 receptors [53]. No connection was reported between patient histopathological features and CRF receptor expression; CRF 1 receptors were located in breast ducts and cancerous implants, with CRF 2 receptors mainly in perineural invasion, and transcript levels of both CRF receptors 1 and 2 did not vary between benign biopsies and cancer tissue from the same tumor [53]. Like in BC tissues, the most abundant receptor type expressed in MCF-7 BC cells was CRF receptor 2 and the transcription of CRF receptors 2 and 1 was respectively down- and up-regulated by estradiol [51]. The activation of CRF receptor 2 increased the migration of MCF-7 cells and potentiated an estrogen-inducing action [51]. Estrogen altered the splicing of CRF receptor 1 in BC cells, changing CRF receptor diversity and disrupting the signaling pathways mediated by CRF [56]. In the estrogen receptor-positive MCF-7 cell line, CRF promoted the activation of kinases and downstream effectors via CRF receptor 1; CRF also augmented the transcription of several genes encoding effectors [56]. Estrogens augmented the mRNA encoding CRF receptor 2 and a splice variant encoding CRF receptor 1 [56]. This variant increase diminished the cell response to CRF and prevented its repressive action on BC cell invasion [56].

A review focusing on the involvement of the CRF peptide family and its receptors in gynecological malignancies (cervical, vulvar, ovarian, endometrial, BC) has been published [57]. CRF peptides mediate cell proliferation, migration, invasion and metastasis as well as regulating the immune response in gynecological tumors [57]. CRF favored MCF-7 BC cell motility and invasiveness and blocked apoptosis, augmented FAK phosphorylation and actin polymerization, favored Cox-1 expression, but not Cox-2 expression, and promoted the synthesis of prostaglandins favoring metastasis [54]. The data suggest that CRF (produced in tumor cells and/or in normal cells of the tumor microenvironment and/or released from nerve terminals and/or arising from the blood stream) promoted the migration of BC cells through actin filament reorganization and the activation of FAK phosphorylation and prostaglandin production via Cox-1. Another study has demonstrated that brain neurons containing CRF controlled anxiety and associated tumor progression [58] and that CRF neurons located in the hypothalamic paraventricular nucleus promoted cancer progression by varying the equilibrium of immune control of cancers [59]. This is an important finding that must be confirmed in BC since it seems that central brain neurons regulate the development of tumors in other tissues.

CRF inhibited MCF-7 cell growth, and this was not related to apoptotic mechanisms [55]. This effect was counteracted with astressin, a non-selective CRF receptor antagonist, and with antalarmin, a selective CRF receptor 1 antagonist [55]. MCF-7 BC cells express both CRF receptor 1 and CRF which is released from tumor cells [55]. CRF blocked the migration of MCF-7 and MDA-MB-231 BC cells via the downregulation of Twist1/Snail1 and the upregulation of E-cadherin [60]. CRF blocked the transforming growth factor β1-mediated migration of MCF-7 cells through CRF receptors 1 and 2, but the inhibition of the migration of MDA-MB-231 cells was mainly mediated via CRF receptor 2 [60]. Moreover, CRF inhibited N-cadherin expression and promoted occludin expression, blocking the EMT in both MDA-MB-231 and MCF-7 BC cells [60]. The data suggest that CRF acts as a tumor suppressor by controlling the transforming growth factor β1-mediated EMT. CRF decreased tumor volume without affecting angiogenesis and increased the action of chemotherapy in 4T1 mouse mammary carcinoma [61]. A study has demonstrated that CRF and urocortin 2 promoted apoptosis in MCF-7 cells by controlling the expression of vitamin D and androgen receptors [52]. CRF downregulated androgen receptor mRNA but upregulated the expression of the androgen receptor protein and promoted nuclear transportation, whereas urocortin 2 inhibited the mRNA production of this receptor but did not affect protein expression [52]. CRF and urocortin 2 augmented the protein expression of vitamin D receptor, which is translocated into the nucleus, and phosphorylated heat shock protein 27, with this being this associated with the nuclear transportation of vitamin D receptors [52]. Further studies need to be undertaken to define the role of CRF and its receptors in BC, pending which one or more agents in this signaling pathway could possibly hold promise for BC.

#### 2.1.5. Endorphins

Endorphins, primarily known as the body’s natural painkillers, are endogenous opioid neuropeptides produced by the central nervous system and the pituitary gland [62]. They play a key role in pain modulation, stress reduction, and feelings of well-being [62]. While endorphins are not classically associated with direct oncogenic or tumor-suppressive roles, emerging evidence suggests they may influence cancer biology indirectly through their effects on the immune system, stress response, and tumor growth [62,63].

A chapter on the roles of endorphins in BC recovery and pathogenesis has been recently published [64]. β-endorphin activated the mitogenic/survival pathways (signal transducer and activator of transcription 3 (STAT3), Akt, ERK, MAPK) in MDA-MB-231 BC cells; however, it seems that β-endorphin controls the stress response and favors innate immunity counteracting BC development [65]. This action mediated by β-endorphin is due to a blockade of sympathetic neuronal action, which augments the synthesis of anti-inflammatory cytokines and the activities of macrophages and natural killer cells [63]; that is, β-endorphin blocks BC development by favoring immune-mediated antitumor defenses [66,67]. Moreover, β-endorphin changed the TME by blocking the synthesis of inflammatory cytokines and catecholamines, leading to the alteration of cell–matrix attachment, angiogenesis, EMT, and DNA repair [63]. Healthy women showed an elevated level of β-endorphin, which was higher in postmenopausal women, whereas in women suffering from BC a lower level of β-endorphin was reported and no differences between postmenopausal and premenopausal women were observed [68].

In a rat model of breast carcinogenesis, β-endorphin-transplanted animals showed a decrease in mammary tumor incidence, malignancy rate, growth and metastasis compared to control animals; in addition, epithelial-to-mesenchymal transition and inflammatory processes were also decreased in the tumor tissues [69]. Moreover, β-endorphin neuron transplants augmented the activities of both macrophages and natural killer cells, decreased plasma levels of inflammatory cytokines, and augmented the plasma levels of anti-inflammatory cytokines [69].

#### 2.1.6. Enkephalins

Enkephalins are endogenous opioid peptides primarily involved in modulating pain and stress responses [70]. They act by binding to opioid receptors (especially δ-opioid and μ-opioid receptors) in the nervous system [70]. However, increasing evidence shows that enkephalins also play roles in cancer biology, influencing tumor growth, immune responses, and possibly metastasis [70]. Some studies show that enkephalins (particularly methionine-enkephalin, also called opioid growth factor (OGF)) can inhibit cancer cell proliferation [70]. OGF binds to the OGF receptor, which regulates cell cycle progression by controlling cyclin-dependent kinase inhibitors (e.g., p16 and p21) [70].

Methionine-enkephalin, but not leucine-enkephalin, promoted the migration of MDA-MB 231 BC cells [71]. However, methionine-enkephalin, through a p21 cyclin-dependent inhibitory kinase pathway, inhibited the proliferation of triple-negative BC cells (MDA-MB-231, BT-20) [72]. Delta opioid receptors are highly expressed in murine and human BC samples, and their stimulation, through the JAK1/2 signaling pathway, was inhibited with delta opioid receptor antagonists [73]. Moreover, the low-fasting pro-enkephalin plasma level observed in postmenopausal middle-aged women has been related with an augmented risk of BC development [74].

#### 2.1.7. Glucagon-like Peptide 1

Glucagon-like peptide 1 (GLP 1) is an incretin hormone primarily produced in the gut (L-cells of the small intestine) and, to a lesser extent, in the brain. It stimulates insulin secretion, inhibits glucagon release, slows gastric emptying and reduces appetite. GLP 1 receptor agonists (e.g., exenatide, liraglutide, semaglutide) are used in treating type 2 diabetes [75].

GLP 1 receptors are expressed in BC cells (KLP-1, MDA-MB-231, MCF-7) [76]. GLP 1 receptor agonists are involved in the suppression and regression of tumors by blocking tumor cell growth, promoting apoptosis, and controlling angiogenesis [77]. Semaglutide, a GLP 1 receptor agonist, decelerated tumor appearance, growth and progression in murine 4T1 BC cells by increasing the acquired anticancer immunity [78]. This agonist augmented the accumulation/maturation of CD11c+ dendritic cells; decreased the number of FoxP3^+^ regulatory T cells; increased tumor infiltration; favored the anticancer phenotype of T cells; and increased the cytotoxic capacity of CD8^+^ T cells [78]. GLP 1 analogs activate the adenosine monophosphate-activated protein kinase and Akt, leading to reversal of the Warburg metabolic switch in BC cells [79]. Thus, through cyclic adenosine monophosphate and adenosine monophosphate-activated protein kinase modulation, GLP 1 analogs altered the metabolism of BC cells (impairing glycolysis), blocking their proliferation [79]. Liraglutide counteracted BC cell growth in obese individuals [80]. This GLP 1 receptor agonist blocked the proliferation of MCF-7 BC cells in obese adipose tissue-derived stem cell-conditioned medium, promoted G0/G1 phase arrest, decreased colony formation and the level of inflammatory mediators, blocked leptin (a carcinogenic adipokine) expression, and augmented mRNA levels of adiponectin (an antineoplastic adipokine) [80]. GLP 1 receptor agonists decreased the risk of developing cancer-related lymphedema in patients following axillary lymph node dissection for BC [81,82] and reduced the risk of basal cell carcinoma and BC but augmented the risk of colorectal cancer [83]. Exendin 4, a GLP 1 receptor agonist, exerted an anti-BC action against BC cells (MCF-7, MDA-MB-231, KLP-1) [84]. This agonist decreased the proliferation of BC cells and DNA synthesis, although apoptosis in BC cells was not observed, and Ki-67-positive proliferative tumor cells and breast tumor weight were reduced in in vivo experiments [84]. This study also reported that the combination of exendin 4 and metformin (used to treat gestational diabetes and type 2 diabetes) is a useful strategy to fight BC progression, since metformin promoted apoptosis in BC cells [84]. Exendin 4 counteracted BC cell growth by blocking nuclear factor κB (NF-κB) activation and decreased the number of BC cells (MCF-7, MDA-MB-231, KLP-1) [76]. Exendin 4 did not promote apoptosis and when MCF-7 cells were transplanted into mice; this GLP 1 receptor agonist decreased tumor size [76]. Exendin 4 blocked NF-κB nuclear translocation and decreased both IκB (inhibitor of kappa B) and Akt phosphorylation [76]. However, another study showed that exendin 4 exerted anticancer effects by promoting apoptosis and inhibiting the growth of MCF-7 BC cells [85], and exendin 4 reduced the expression of Akt, caspase 9 and metalloproteinase 2, whereas it increased the expression of caspases 3, 8 and 10, p53, phosphatase and tensin homolog (PTEN), tissue inhibitor of metalloproteinase (TIMP) 1 and 2, and poly-ADP ribose polymerase (PARP) in MCF-7 BC cells [85].

Liraglutide, an analog of GLP 1, increased the expression of this peptide in BC cells (MDA-MB-231, MDA-MB-468) and tissues derived from rodents bearing 4T1 cell inoculation [86]. This analog, after activating GLP 1 receptors, accelerated BC in vitro and in vivo via the NADPH oxidase 4/reactive oxygen species/VEGF signaling pathway, whereas exendin (9–39), a GLP 1 receptor antagonist, blocked the effects mediated by liraglutide [86]. This means that liraglutide favored the progression of triple-negative BC cells. This finding was also reported in another study: liraglutide favored growth promotion and increased ATP-binding cassette transporter expression (suggesting increased EMT and drug resistance) in the triple-negative BC cell line MDA-MB-231 [87]. However, a study based on 52 trials reported that treatment with GLP 1 receptor agonists for diabetes and obesity did not augment the risk of BC development [88]. Other studies concluded that the use of liraglutide did not increase the risk of BC [89] and that the administration of GLP 1 analogs was not related to an increase BC risk in women suffering from type 2 diabetes [90]. Another study stated that the detection of BC gradually augmented weight loss categories with GLP 1 receptor agonists, in particular in those women achieving >10% weight loss [91]. The authors concluded that important weight loss due to treatment with GLP 1 receptor agonists could serve to detect BC among obese women suffering from type 2 diabetes.

#### 2.1.8. Gonadotropin-Releasing Hormone/Luteinizing Hormone-Releasing Hormone

Gonadotropin-releasing hormone (GnRH), also known as luteinizing hormone-releasing hormone (LHRH), and its receptors are expressed in several tumor types, including prostate cancer, BC, endometrial cancer and ovarian cancer. GnRH analogs are explored for anticancer therapy because they can inhibit tumor growth, especially in hormone-sensitive cancers [92]. Goserelin (Zoladex), a GnRH analog, has been approved by the FDA for the palliative treatment of advanced BC in both post- and pre-menopausal women [12]. GnRH receptor 2 analogs exerted pro-apoptotic, antiproliferative and antimetastatic actions against BC and other cancers (e.g., ovarian, endometrial, prostate) [92,93,94]. The co-administration of Src/FAK inhibitors and GnRH receptor antagonists (degarelix) counteracted BC growth and metastasis and augmented animal survival, whereas the use of leuprorelin (a GnRH receptor agonist) favored tumor progression and controlled gene expression associated with tumor progression [95]. GnRH receptor mRNA level was higher in patients with triple-negative BC than in patients with BC expressing human epidermal growth factor receptor (HER) 2 [96]. Moreover, patients with a high expression of GnRH receptors showed a better disease-free survival than those showing a lower expression and, importantly, the activation of the GnRH receptor blocked cell proliferation and metastasis, promoted apoptosis, and increased the protein expression of GnRH receptors in triple-negative BC cells [96].

The targeting of triptorelin-conjugated dextran-coated magnetite nanoparticles as a targeted probe in positive GnRH receptor tumor cells in magnetic resonance imaging has been reported [97]. This opens the door for imaging, diagnosis and treatment of cancers expressing GnRH receptors [97,98]. Moreover, the characterization of a recombinant immunotoxin (GnRH-DNA fragmentation factor 40) for targeted therapy of BC cells (SKBR-3, MDA-MB-231, MCF-7) expressing GnRH receptors has been published [99]. This immunotoxin promoted apoptosis in BC cells and, in addition, blocked the invasive capacity of MDA-MB-231 cells [99].

As indicated above, LHRH plays an important role in cancer biology, especially in hormone-sensitive cancers [100]. Studies show LHRH analogs can inhibit tumor growth by reducing sex hormone levels [101], induce apoptosis in LHRH-receptor-positive cancer cells and be effective as part of combination therapies (e.g., with chemotherapy or targeted agents) [101]. Triple-negative BC cells overexpress LHRH receptors; therefore, LHRH-conjugated drugs can be adopted to fight BC [102]. Thus, conjugated drugs (LHRH-conjugated paclitaxel; LHRH-conjugated prodigiosin) showed a higher anticancer effect against triple-negative BC cells than unconjugated drugs (prodigiosin, paclitaxel) [102,103], and an inhibition of the growth of BC cells has been reported in in vivo and in vitro experiments [102]. Pt-Mal-LHRH, a new chemotherapeutic compound, decreased triple-negative BC tumor growth (4T1, MDA-MB-231) in vivo [104]. The fusion of LHRH to its pore-forming domain (BinBc) blocked the proliferation of MCF-7 BC cells, but this compound did not affect human fibroblasts (Hs68) [105]. BinBc alone did not affect the proliferation of both cells, and LHRH-BinBc promoted the efflux of lactate dehydrogenase and induced apoptosis in BC cells via the activation of caspase 8; LHRH-BinBc was mainly located on the cell surface of both MCF-7 and Hs68 cells [105]. Other studies have reported the synthesis of radiolabeled technetium- and thenium-LHRH conjugated to detect and target BC cells overexpressing LHRH receptors [106], and an LHRH receptor-targeted and tumor microenvironment-responsive nanoparticle system (LHRH-DCMs) to deliver selectively chemotherapeutic drugs to triple-negative BC cells has been published [107]. GnRH/LHRH analogs should be harnessed for use as a therapeutic strategy for BC.

#### 2.1.9. Kisspeptin

Kisspeptin is a peptide that plays a crucial role in regulating the reproductive system, particularly by controlling the release of GnRH from the hypothalamus [108]. First identified as a metastasis suppressor in melanoma (thus the name “kisspeptin” from Kiss-1) [109], kisspeptin exerts an antimetastatic action in some cancers (brain, lung, colon), but in BC this peptide promotes aggressiveness and aggravates BC prognosis [110]. Kisspeptin 1/kisspeptin 1 receptor and MMP 9 expressions were higher in BC samples than in non-cancerous tissues placed near the breast tumor, and a positive correlation was observed between MMP and kisspeptin 1 and between aromatase expression and kisspeptin 1 receptor [111]. Kisspeptin 1/kisspeptin 1 receptor did not correlate with Ki-67 and cyclin D1 levels and a higher expression of kisspeptin 1 receptor was observed in estrogen receptor-negative cases than in estrogen receptor-positive cases in BC patients with lymph node metastasis [111]. Kisspeptin binds to the G protein-coupled receptor GPR54 (kisspeptin 1 receptor), which is highly expressed in BC, and this overexpression could be used for drug delivery (e.g., doxorubicin-loaded 228-K3-EG8-liposome) [8]. This strategy blocked BC cell proliferation and augmented the median survival time in mice with BC [8]. Kisspeptin promoted GPR54 mRNA expression in both MCF-7 and SKBR3 BC cells, whereas the induction of aromatase (CYP19A1) was observed in MCF-7 cells but not in SKBR3 cells [112]. Kisspeptin 1 receptor mediated triple-negative BC cell invasion and, compared with normal breast samples, kisspeptin/kisspeptin 1 mRNA/kisspeptin 1 protein were upregulated in triple-negative BC cells [113]. Moreover, kisspeptin 1 receptor signaling favors drug resistance by augmenting the expression of the efflux drug transporter (BC resistance protein) and by favoring the activity/transcription of the receptor tyrosine kinase, AXL [113]. BC resistance protein and AXL transcripts were elevated in triple-negative BC cells when compared with normal breast samples, and triple-negative BC tumors expressing kisspeptin 1 receptor also expressed AXL and BC resistance protein [113]. Kisspeptin favored the formation of invadopodia by controlling the cell cytoskeleton and induced cell invasion (triple-negative BC cells) and metastasis [110]. The kisspeptin 1 receptor mediated the formation of invadopodia in BC cells through the β-arrestin 2/ERK 1/2 signaling pathway (Src-independent) and activated invadopodia proteins (membrane type I matrix metalloproteases, cofilin, cortactin) [114]. Kisspeptin 1 receptor depletion decreased the mesenchymal phenotype and invasiveness of triple-negative BC cells [114]. The matrix protein fibulin 3 favors kisspeptin 1 receptor-induced triple-negative BC cell invasion: the fibulin 3 gene is amplified in these cells; the plasma fibulin 3 level is higher in patients with triple-negative BC than in healthy individuals; and the activation of the kisspeptin 1 receptor augmented both the release and expression of fibulin 3 [115]. Moreover, fibulin 3 controlled triple-negative BC metastasis in a rodent experimental model of metastasis, and signals downstream of kisspeptin 1 receptor favored triple-negative BC cell invasion by activating the MAPK pathway and MMP 9 [115]. The authors concluded that fibulin 3 is a promising biomarker for triple-negative BC progression, invasion and metastasis. Kisspeptin 1 receptor mediates tumor growth and metastasis in vivo and nucleotide biosynthesis and glutaminolysis by augmenting the levels of glutaminase and c-Myc, which are involved in the metabolism of glutamine [116]. Kisspeptin 1 is needed for transforming growth factor-β-induced triple-negative BC cell invasion and, in fact, kisspeptin 1 knockdown expression inhibited the invasion mediated by this transforming growth factor (which favors tumor development and metastasis in BC) and the expression of MMP 9 [117]. Kisspeptin-10 (KP-10, the shortest active kisspeptin peptide) promoted BC invasion via the activation of the MAPK/ERK pathway, and a high level of kisspeptin 1 has been associated with the lymph node-positive grade [117]. KP-10 favored the migration and invasion of BC cells (without estrogen receptors) by cross-talking with EGFR (epidermal growth factor receptor), through a β-arrestin 2-dependent process [114]. MDA-MB-231 and BT-20 BC cells expressed kisspeptin 1 receptor mRNA, and KP-10 promoted migration in MDA-MB-231 cells but not in BT-20 cells [118].

The kisspeptin 1 receptor is highly expressed in patients with triple-negative BC; this receptor is involved in the malignant transformation of BC epithelial cells, and a higher level of kisspeptin has been observed in patients with triple-negative BC than in healthy individuals [116].

An inverse correlation of kisspeptin 1 and kisspeptin 1 receptor expression in African American women suffering from triple-negative BC has been reported: kisspeptin 1 receptor was higher in non-triple-negative BC than in other groups; kisspeptin 1 protein expression was higher in receptor-negative and triple-negative BC than in other populations; kisspeptin 1 receptor was marginally negatively associated with tumor size but positively correlated with disease-free survival and lymph node positivity [119]. Kisspeptin 1 receptor mRNA expression was higher in women with advanced stages of BC (stage III) than in BC patients showing stage II; a correlation was reported between kisspeptin 1 receptor mRNA expression and lymph node metastasis and tumor size; kisspeptin 1 receptor mRNA expression was more highly expressed in estrogen receptor-negative cases than in estrogen receptor-positive subjects and in progesterone receptor-negative cases than in progesterone receptor-positive ones [120]. An overexpression of kisspeptin 1 receptor mRNA was observed in patients expressing human epidermal growth factor receptor 2 (HER2) and in triple-negative BC subjects [120]. A study performed in patients with BC showed that less elevated kisspeptin expression was associated with a negative prognostic factor for overall survival, axillary lymph node status, metastatic propensity, advancing tumor stage, and advanced grade [121]. Kisspeptin levels were higher in BC than in normal samples, and kisspeptin expression was higher in non-metastatic cases than in metastatic ones [121]. Astrocytes promote the metastatic transformation of circulating BC cells in the brain via the release of the chemokine CXCL12; these cells favor brain invasion of the circulating BC cells by increasing autophagy signaling pathways through the chemokine CXCL12-microRNA 345-kisspeptin 1 axis [122]. The rs5780218 polymorphism of the kisspeptin 1 gene has been related to an increased risk of BC development [123].

Despite the numerous data previously mentioned demonstrating the oncogenic action of kisspeptin, other studies have shown that kisspeptin exerts an anticancer action. Thus, kisspeptin blocked metastatic SKBE3 BC cell growth, migration and metastasis through the activation of eukaryotic translation initiation factor 2α kinase 2 (EIF2AK2) [124]. Kisspeptin decreased the proliferation of MCF-7 (estrogen receptor-positive) and MDA-MB-231 (estrogen receptor-negative) cells, favored the synthesis of interleukin-8 in both cell types, decreased the motility of MCF-7 cells and promoted the motility of MDA-MB-231 cells [125]. Kisspeptin 1 counteracted angiogenesis of BC brain metastasis [126], and KP-10 blocked the migration of BC cells (MDA-MB-231, MDA-MB-157) in vivo and in vitro by controlling the EMT, promoted apoptosis, decreased the motility of both BC cells, blocked the formation of intratumoral blood microvessels, inhibited tumor growth in vivo and improved the survival rate of experimental animals [127]. KP-10, via the activation of the Smad signaling pathway, blocked the Warburg effect and favored mitochondrial injury in MDA-MB-231 BC cells and, compared with non-cancerous tissues, mRNA and protein levels of pyruvate dehydrogenase kinase, pyruvate kinase and hexokinase 2 were higher in BC tissues [128]. Melatonin, through the expression of kisspeptin, blocked triple-negative BC metastasis (HCC-70, MDA-MB-231) but melatonin did not affect proliferation in these cells [129]. Kisspeptin expression was regulated by melatonin through the expression/transcriptional activation of GATA binding protein 3, and kisspeptin silencing enfeebled melatonin blockade of BC cell invasiveness [129]. Moreover, stromal-derived factor-1 favored the invasion and EMT of MCF-7 BC cells, and both processes were blocked with KP-10 by downregulating the expression of CXC-motive-chemokine receptor 4 [130]. BC cell invasion was inhibited with antibodies directed against stromal-derived factor-1, and treatment of MCF-7 cells with KP-10 reduced the protein expression of CXC-motive-chemokine receptor 4 [130].

#### 2.1.10. Oxytocin

Oxytocin, a neuropeptide hormone best known for its roles in childbirth, lactation, social bonding, and stress regulation, also has emerging relevance in cancer biology [131]. Oxytocin inhibits tumor growth, often through the oxytocin receptor [132]. Mechanisms include inhibition of cell proliferation, induction of apoptosis, reduction in cancer cell migration and invasion and suppression of angiogenesis [133,134].

The use of peptide-based tracers directed against upregulated oxytocin receptors is a promising therapeutic strategy to diagnose and treat BC [135]. Oxytocin exerted antiproliferative actions in BC cell lines (MDA-MB-231, MCF-7, T47D) [136]. In contrast, an overexpression of oxytocin receptors has been associated with mammary hyperplasia and tumorigenesis via the activation of the prolactin/p-STAT5 pathway [137]. In this study, the prolactin inhibitor bromocriptine counteracted oxytocin receptor-driven cancer growth [137]. Oxytocin expression is higher in BC subjects than in healthy individuals [138], and the expression of oxytocin receptors is higher in adjacent BC tissues, followed by normal and tumor tissues [139]. The level of this receptor was high in MDA-MB-231 cells, and a high expression of oxytocin receptors has been associated with an enhanced metastasis capacity; in fact, metastasized tumors showed a higher expression of oxytocin receptors than the corresponding primary tumors, and a high expression of oxytocin receptors increased tumor cell migration, via the ERK1/2-RSK-rpS6 signaling pathway, and decreased survival in triple-negative BC individuals [139]. However, it has been reported that oxytocin-associated genes are dysregulated in BC tissues; for example, a downregulation of the expression of oxytocin receptors has been reported [140]. In addition, oxytocin receptor expression was lower in BC tissues than in normal tissues from the same subject, and oxytocin receptor mRNA level was lower in estrogen receptor-negative BC samples compared to estrogen receptor-positive BC tissues [141]. Previous contradictory findings must be investigated in depth. Estradiol augmented the oxytocin receptor mRNA level in MCF-7 BC cells but not in MDA-MB-231 cells (estrogen receptor-negative) [141]. Additionally, an increase in insulin-regulated aminopeptidase (IRAP, an enzyme that cleaves oxytocin) activity has been detected in BC tissues, and in women with BC treated with neoadjuvant chemotherapy, IRAP activity was augmented in both postmenopausal and premenopausal women [142].

Oxytocin plays an important role in the resilience of individuals suffering from BC [143]. Oxytocin also plays a significant role in the success of exercise training on BC; thus, interval exercise training, through the release of oxytocin (plasma levels are higher in trained animals than in untrained mice), decreased the ERK and PI3K/Akt axis, reducing tumor weight/volume in a rodent experimental model of BC [144]. Moreover, compared with untrained mice, the expression of genes involved in cancer cell proliferation (*Akt*, *mTOR*, *PI3KR2*) was lower in trained animals and oxytocin-treated animals [144]. The expression of genes related to cell apoptosis (*Bax*, *caspase 3*) was higher in cancer tissues, and phosphorylated ERK/Akt were reduced in the trained animals and in those treated with oxytocin [144].

### 2.2. Oncogenic Peptides

#### 2.2.1. Adrenomedullin 2

AM2 is emerging as a relevant factor in cancer biology, although it has been less extensively studied than AM. Its roles are multifaceted and include promotion of survival, proliferation, angiogenesis and resistance to apoptosis [145]. AM2 promoted BC cell growth, migration and invasion; its expression was increased in BC samples, and the level of AM2 has been correlated with Ki-67 expression and lymph node metastasis [146]. BC cell growth, migration and invasion were blocked with anti-AM2 antibodies, and this strategy also reduced tumor growth and lung metastasis of 4T1 BC cells in vivo [146]. Moreover, AM2 promoted Src kinase phosphorylation, triggering c-Myc transcription, which controls the expression of genes encoding ribosomal constituents; accordingly, AM2 favored BC cell invasion and metastasis by increasing protein translation and ribosome biogenesis through the c-Myc/Src signaling pathway [146]. Further investigation is necessary to further understand and harness anti-AM2 antibodies for BC treatment.

#### 2.2.2. Endothelin

Endothelins are a family of potent vasoconstrictive peptides, with endothelin 1 being the most studied. While originally identified for their role in vascular homeostasis, they are now recognized as important players in cancer development and progression [147,148]. Endothelin 1 increased MDA-MB-231 invasiveness [149], and an endothelin 1-enriched tumor phenotype has been related with a higher risk for BC recurrence [150]. The stimulation of MCF-7 and MDA-MB-231 cells by endothelin 1 favored the activation of Akt, and the silencing of endothelin receptor 1 promoted apoptosis in BC cells [150]. Bosentan, a dual non-selective endothelin receptor A/B antagonist, inhibited the proliferation and migration of MCF-7 cells mediated by endothelin 1 [151]. Endothelin B receptor isoforms have been reported in BC cells, and in knocking down the endothelin B receptor gene in these cells, invasiveness was altered [152]. The endothelin receptor B is involved in tumorigenesis and metastasis; its expression is lower in primary BC than in metastatic tumors, and its expression has been related to poor survival and lymph node metastasis [153]. In triple-negative BC cells an upregulation of the endothelin receptor B has been reported, and the silencing of this receptor reduced the proliferation, migration and invasion of BC cells (BT-549, MDA-MB-231), increased apoptosis and retarded the growth of implanted tumors in experimental animals [153]. This silencing also reduced extracellular regulated protein kinase phosphorylation and favored the mesenchymal-to-epithelial transition mechanism in MDA-MB-231 BC cells [153]. Intermittent hypoxia promoted murine 4T1 BC development (proliferation and migration) via endothelin A receptors and favored tumor growth in vivo, and treatment with macitentan (a dual endothelin A/B receptor antagonist) prevented tumor development [154]. This dual antagonist also enhanced the efficacy of ado-tratuzumab emtansine against brain metastasis from HER2-positive BC cells [155], and macitentan sensitized experimental BC (MDA-MB-231) brain metastases to paclitaxel in mice [156]. The co-administration of macitentan and paclitaxel decreased tumor cell proliferation and increased overall survival, and a decrease in marked apoptosis was observed [156].

The upregulation of endothelin 1 may predict the risk of developing chemotherapy-induced cardiotoxicity in women suffering from BC [157]. BC is related to left ventricular hypertrophy and elevated endothelin 1 signaling because BC cells favor cardiomyocyte hypertrophy via the release of endothelin 1 [158]. In this sense, endothelin receptor blockers counteracted BC-induced cardiac remodeling; one of these blockers, atrasentan, improved cardiac functions and reduced cardiac remodeling in a rodent model of BC [159]. Moreover, an endothelin 1 genetic polymorphism as a predictive marker for bevacizumab in metastatic BC has been suggested: the single-nucleotide polymorphisms rs5370 in endothelin 1 could serve to identify patients who are unlikely to gain any advantage from bevacizumab [160].

#### 2.2.3. Gastrin-Releasing Peptide

Gastrin-releasing peptide (GRP) plays a significant role in gastrointestinal and central nervous system functions, such as stimulating gastrin release and regulating circadian rhythms [161]. However, GRP and its receptor are also implicated in cancer development and progression, particularly in tumors of neuroendocrine origin [162,163]. This peptide was highly expressed in BC patients with lymph node metastasis; patients with higher expression of GRP had shorter survival times [164]. The overexpression of this peptide has been related to an enhanced invasive capacity of tumor cells; in fact, a knockdown of GRP reduced this capacity in MCF-7 BC cells [164]. Moreover, an overexpression of the GRP receptor has been reported in BC cells, and this means that this receptor is a potential theranostic target in BC (e.g., using the theranostic pair [^55^Co]Co- and [^177Lu^]Lu-DOTA-RM26 (a GRP receptor antagonist) which reduced the viability of tumor cells; daunorubicin-containing peptide-drug conjugate) [7,165,166,167,168,169,170,171,172].

#### 2.2.4. Neurokinin A

Neurokinin A is a member of the tachykinin peptide family, which also includes substance P and neurokinin B. Neurokinin A is best known for its roles in smooth muscle contraction, pain transmission, and inflammation; recent research has shown that it may also play a role in cancer development and progression [173,174]. Neurokinin A promotes the proliferation of BC cells expressing neurokinin receptor 2, and an overexpression of neurokinin receptors 1 and 2 was observed in metastatic BC cells compared to non-metastatic ones [175,176]. Neurokinin receptor 2 mediated the proliferation of BC cells but this receptor was not involved in the proliferation of normal cells [177]. Moreover, neurokinin A promoted the migration and invasion of BC cells and hence augmented the aggressiveness of metastatic BC cells [178]; it increased the expression of neurokinin receptors 1 and 2 in metastatic BC cells and favored the secretion of a bradykinin precursor (high-molecular-weight kininogen compound) that mediates tumorigenic effects [178]. Neurokinin receptor 2 antagonists inhibited the proliferation of BC cells [177].

#### 2.2.5. Neuromedin

Neuromedins are a family of peptides; they often function as neurotransmitters or neuromodulators in the nervous system but can also have roles in various physiological processes throughout the body [179]. Neuromedins have been implicated in cancer biology due to their ability to influence cell proliferation, migration and invasion, angiogenesis and apoptosis resistance [180]. Neuromedin B is a growth and pro-angiogenic factor [181]. The neuromedin B receptor antagonist PD168,368 inhibited tumor growth and angiogenesis, mediated by neuromedin B, in in vivo and in vitro experiments [180]. This antagonist promoted apoptosis and cell cycle arrest in MDA-MB-231 cells, blocking the migration/invasion and decreasing the EMT (by vimentin downregulation and E-cadherin upregulation) of these BC cells [180,181]. PD168,368 also blocked the metastasis of BC cells in vivo [181]. Hypoxia increased the levels of neuromedin B receptor mRNA and protein in BC cells via a mechanism dependent on hypoxia-inducible factor (HIF) 1α [182].

Neuromedin U expression is upregulated in BC tissue when compared to healthy breast tissue, and this expression has been associated with poor outcome in breast tumors showing a strong expression of neuromedin U receptor 2 [183]. Neuromedin U expression affected molecules involved in Wnt receptor signaling: a downregulation of canonical Wnt targets (e.g., Myc) and an increased activation of the Wnt/planar cell polarity effector RAC1 were reported [183]. Moreover, the expression of neuromedin U favored a motile phenotype in neuromedin U receptor 2-positive SKBR3 cells but not in neuromedin U receptor 2-negative Hs578T cells [183]. The data suggests that neuromedin U promoted the progression of BC cells expressing neuromedin U receptor 2. Overexpression of neuromedin U in HER2-positive BC cells augmented glycolysis because the activity of pyruvate dehydrogenase kinase activity was increased, and this was also observed in HER2 drug-resistant cells [184]. This overexpression led to the upregulation of the EMT markers and to an increased interleukin-6 release; all previous mechanisms were related to cancer stem cell phenotype [184]. Thus, neuromedin U in HER2-overexpressing BC increased the resistance to HER2-targeted drugs via conferring cancer stem cell characteristics and cancer stem cell phenotype expansion [184]. Overexpression of neuromedin U in drug-sensitive cells promoted resistance to HER-targeting drugs; the peptide increased HER-2 and EGFR expressions along with drug resistance, and neuromedin U attenuation weakened cancer growth and metastasis [185]. In this sense, neuromedin U has been suggested as a therapeutic target and a candidate biomarker to predict and overcome resistance to HER-tyrosine kinase inhibitors; it is also a useful tool to improve HER-targeted drug efficacy [185]. Moreover, the resistance to HER2-targeted antitumor drugs has been related to immune evasion in tumor cells: neuromedin U in HER2-overexpressing BC cells increased resistance to anticancer immune responses [186]. The neuromedin B/neuromedin B receptor system could possibly serve as a target for BC treatment.

#### 2.2.6. Neuropeptide Y

Neuropeptide Y is a peptide neurotransmitter widely expressed in the central and peripheral nervous systems [187]. It is involved in various physiological processes like appetite regulation, stress response, angiogenesis, and cell proliferation [187,188]. Neuropeptide Y can influence cancer cell proliferation and survival [188]. It has been shown to promote tumor growth in certain cancers by acting through its receptors, mainly Y1, Y2, and Y5 receptors, which are G protein-coupled receptors [189,190]. Many cancer types overexpress neuropeptide Y receptors, especially Y1 and Y5, which can mediate oncogenic signaling pathways that enhance cancer cell proliferation, migration, invasion and angiogenesis [191,192].

The neuropeptide Y/neuropeptide Y receptor system promotes BC cell proliferation, migration, invasion and metastasis and angiogenesis, whereas neuropeptide Y receptor antagonists inhibit all these effects and favor the death of tumor cells [188]. Neuropeptide Y and neuropeptide Y receptors 1 and 5 are highly expressed in BC [191], and neuropeptide Y receptor 5 antagonists (CGP71,683A) inhibited both BC cell growth and migration and also promoted the death of BC cells expressing neuropeptide Y receptor 5 [193]. Neuropeptide Y receptor 1/5 mRNA levels were augmented by hypoxia-inducible factors, which sensitized both receptors to neuropeptide Y activation, promoting the proliferation and migration/invasion of BC cells (MCF-7, MDA-MB-231) [191]. Compared with normoxia conditions, a higher decrease in spheroid growth/invasion, MAPK signaling, and cell proliferation, migration and invasion was observed in hypoxia conditions after the administration of neuropeptide Y receptor 1/5 antagonists [191]. MCF-7 BC cells were less invasive when neuropeptide Y receptor 5 was blocked [191]. The authors concluded that neuropeptide Y receptor 1 protein levels are related with adverse outcomes and that neuropeptide Y receptor 5 protein levels and colocalization with hypoxia conditions are associated with advanced cancer [191].

A high serum neuropeptide Y receptor 1 level is positively correlated with clinical stage and lymph node metastasis, and BC patients expressing neuropeptide Y receptor 1 had a shorter cancer-specific survival than those individuals without this expression [194]. Mortality rate was correlated with the expression of HER2 in both neuropeptide Y receptor 1-positive/negative groups of BC patients [194]. Moreover, the high expression of neuropeptide Y receptor 1 has been related to perineural invasion, advanced stages and lymph node metastasis [188,194,195]. Tamoxifen promoted the loss of neuropeptide Y receptor 1 in MCF-7 BC cells in in vivo experiments [196]. Neuropeptide Y receptor 1 gene expression was augmented when estrogen receptor-positive BC cells/experimental animal models were treated with estrogens, whereas the expression of neuropeptide Y receptor 1 decreased in estrogen receptor-positive BC cells resistant to endocrine treatments (fulvestrant, tamoxifen, estrogen deprivation) in vivo and in vitro [197]. In primary BC tumors and BC-derived metastasis, an overexpression of neuropeptide Y receptor 1 has been reported, but in normal BC samples the most expressed receptor was neuropeptide Y receptor 2 [198]. Neuropeptide Y receptor 1 mediated the inhibitory effect of neuropeptide Y on estradiol-activated growth of estrogen receptor-positive BC cells, and the expression of neuropeptide Y receptor 1 has been suggested to be a biomarker to predict better survival and endocrine sensitivity in estrogen receptor-positive BC subjects [197]. A higher neuropeptide Y receptor 1 gene expression is correlated with a better overall survival and relapse-free survival in estrogen receptor-positive BC patients [197]. Neuropeptide Y receptor 5 agonists augmented the level of VEGF in 4T1 BC cells, but this did not occur when neuropeptide Y receptor 1 or 2 agonists were administered [199]. Moreover, neuropeptide Y receptor 5 agonists favored the secretion of VEFG from BC cells, promoting angiogenesis [199].

Neuropeptide Y receptors have been suggested as molecular targets in BC treatment, and, in this sense, neuropeptide Y analogs have been proposed as specific BC-targeting agents [200]. The overexpression of neuropeptide Y receptors in BC cells allows the use of designed compounds for breast tumor imaging (e.g., heterobivalent dual-target peptide for neuropeptide Y and integrin α_v_β_3_ receptors; ^18^F-labeled triazolyl-linked argininamides, neuropeptide Y analogs labeled with a positron emitter ^68^Ga; 99mTc-labeled neuropeptide Y short analog) [201,202,203,204,205] and BC treatment (Y1 L-KGRR-FF-IR: an enzyme-responsive precursor based on the neuropeptide Y receptor 1 ligand; chlorin e6 delivery system; neuropeptide Y-decorated gold nanoclusters) [206,207,208,209]. Y1 L-KGRR-FF-IR generated apoptosis in BC cells and decreased BC tumor volume in experimental animals [206], and the chlorin e6 delivery system suppressed BC tumors overexpressing neuropeptide Y receptor 1 in experimental animals [207]. Neuropeptide Y-decorated gold nanoclusters blocked protein synthesis via the MAPK pathway and promoted apoptosis in MCF-7 BC cells [208]. A prodrug (doxorubicin-P18) based on neuropeptide Y analog showing cancer microenvironment responsiveness has been developed to fight triple-negative BC cells: this prodrug exerted a higher suppression of tumor growth and metastasis than the administration of free doxorubicin [210]. In conclusion, the neuropeptide Y/neuropeptide Y receptor system has conflicting roles in BC; further investigations are necessary to define its role in cancer pathogenesis, pending which analogs/antagonists could be used as appropriate for BC treatment.

#### 2.2.7. Neurotensin

Neurotensin is a 13-amino-acid peptide found in the central nervous system and the gastrointestinal tract [211]. Many cancers, including pancreatic, colorectal, prostate, breast, and lung cancers and glioma, overexpress neurotensin and/or its receptor [212,213,214]. Neurotensin acts as a growth-promoting factor in various cancers through neurotensin receptor 1, activating oncogenic pathways that support tumor growth, invasion, and survival [215]. Its involvement in cancer progression makes it a promising target for novel anticancer therapies and diagnostic tools [215].

Neurotensin and neurotensin receptor 1 are upregulated in BC [216], and plasma pro-neurotensin has been associated with BC development [217]. Neurotensin, through neurotensin receptors, promoted oncogenic mechanisms in BC (cell proliferation, migration, invasion and metastasis) and an anti-apoptotic action, whereas neurotensin receptor antagonists/neurotensin receptor 1 silencing inhibited all previous actions [218,219,220]. A relationship between the appearance of metastasis and the expression of neurotensin receptor 3 has been reported [221]. Moreover, the administration of the neurotensin receptor 1 antagonist SR48,692 or the silencing of this receptor blocked tumor growth in experimental mice xenografted with MDA-MB-231 BC cells [220]. The neurotensinergic system also favored the overexpression of HER2/3 and EGFR in BC and, in an experimental model of BC, the growth of BC cells overexpressing neurotensin/neurotensin receptor 1 was blocked with lapatinib (an HER2/EGFR tyrosine kinase inhibitor) or metformin [216]. The activation of HER2-3 and EGFR by the neurotensin/neurotensin receptor 1 system renders breast tumors aggressive, and the administration of neurotensin receptor 1 antagonists blocked the adherence and migration/invasion of BC cells [216]. Moreover, the overexpression of the neurotensinergic system has been correlated with higher aggressiveness, worse sensitivity to chemotherapeutic drugs, tumor size, poor prognosis, and increased relapse risk [216,219]. In normal epithelial breast cells, neurotensin is expressed and upregulated by estrogens; the peptide is also expressed in the ductal and invasive components of invasive ductal breast carcinomas, and a high neurotensin receptor 1 expression has been related to tumor size, number of metastatic lymph nodes, and SBR (Scarff–Bloom–Richardson) grade [222]. Thus, the neurotensinergic system is involved in ductal BC progression.

Neurotensin receptor 1 has been observed in the cytoplasm or nucleus of primary breast tumors, and it seems that this distribution is mutually exclusive [223]. The overexpression of cytoplasmic neurotensin receptor 1 was associated with higher pT (pathological) stage and higher tumor grade, whereas the nuclear location was correlated with lower pT stage, low Elston and Ellis grade and estrogen receptor positivity [223]. Compared to nuclear localization, the cytoplasmic expression of neurotensin receptor 1 has been related to shorter 10-year metastasis-free intervals [223].

#### 2.2.8. Substance P

Substance P is an 11-amino-acid neuropeptide belonging to the tachykinin family [224]. It primarily binds to neurokinin receptor 1, a G protein-coupled receptor [224]. It is widely known for its roles in pain transmission, inflammation, and stress responses.

Substance P can promote tumor cell proliferation by activating neurokinin receptor 1 [224]. The activation of this receptor triggers signaling pathways like MAPK/ERK and PI3K/Akt, which are involved in cell growth and survival [225]. Many types of cancer cells overexpress neurokinin receptor 1, making them responsive to substance P stimulation [226]. A higher expression of pre-protachykinin A and neurokinin receptor 1 has been reported in BC cells and malignant BC biopsies, compared with that found in normal mammary epithelial cells and benign BC biopsies, whereas in malignant and normal cells a high expression of neurokinin receptor 2 was observed [177]. Substance P favored the migration and invasion of BC cells; thus, the aggressiveness of metastatic BC cells was increased [178]. Moreover, substance P increased the expression of neurokinin receptor 1 in metastatic BC cells (but not that of neurokinin receptor 2) and favored the secretion of a bradykinin precursor (high-molecular-weight kininogen compound) that mediated tumorigenic effects [178]. The release of substance P from sensory nerve cells favored breast tumor growth, invasion and metastasis, and the administration of aprepitant (a neurokinin receptor 1 antagonist) blocked BC growth and metastasis [227]. Moreover, a higher innervation was found in highly metastatic murine mammary tumors than in less metastatic tumors, and an enhanced lymph node metastatic spread was reported in patient tumors with elevated substance P [227].

Neurokinin receptor 1 antagonists inhibited the proliferation of BC cells [177]. In this sense, a study has reported that the neurokinin 1 receptor antagonist aprepitant is a promising candidate for BC treatment [228]. In this study, MT-3, BT-474, MCF-7 and MDA-MB-231 BC cell lines were studied, and three neurokinin 1 receptor antagonists (L-733,060, L-732,138, aprepitant) were tested. The most important findings of this study were the following: BC cells express mRNA for neurokinin receptor 1; this receptor is overexpressed in BC cells; neurokinin receptor 1 mediates the viability of BC cells; substance P promotes the proliferation of BC cells; neurokinin receptor 1 antagonists, via neurokinin receptor 1, block the mitogenesis of BC cells mediated by substance P, and neurokinin receptor 1 antagonists promote the death of BC cells by apoptotic mechanisms [228]. Moreover, substance P and neurokinin receptor 1 were observed in all human BC samples studied [228]. The authors concluded that neurokinin receptor 1 is a promising target to fight BC by administering neurokinin receptor 1 antagonists such as the drug aprepitant [228]. Moreover, in a review focused on the involvement of the substance P/neurokinin receptor 1 system in triple-negative BC, the authors concluded that neurokinin receptor 1 antagonists, including aprepitant, exerted antiproliferative, antimetastatic and apoptotic effects against triple-negative BC cells overexpressing neurokinin receptor 1 and, in addition, these antagonists decreased the tumor volume of triple-negative BC cells in experimental animals [229].

#### 2.2.9. Vasoactive Intestinal Peptide

Vasoactive intestinal peptide (VIP) is a peptide that functions as a neurotransmitter and hormone [230]. VIP acts primarily through G protein-coupled receptors: VPAC1, VPAC2, and PAC1 receptors [231]. Many cancer cells and tumor tissues have been shown to overexpress VIP receptors, especially VPAC1 [232]. This overexpression can be exploited for diagnostic imaging and targeted therapy [232]. VIP can act as a growth factor in some cancers by activating signaling pathways that promote cell proliferation. It may stimulate tumor growth through activation of cAMP pathways and downstream signaling cascades (e.g., PKA, MAPK) [233]. However, the effect of VIP can vary depending on tumor type; in some contexts, it might inhibit proliferation or induce differentiation [234,235].

VIP receptor 2 is involved in BC cell proliferation and migration [236]. It has been shown that VIP receptor 2 dimerizes, that monomers of this receptor interact with each other via transmembrane domains 3–4, and that these domains prevent the dimerization of VIP receptor 2 [236]. Moreover, BC cells expressing transmembrane domains 3–4 blocked lymph node metastasis and tumor growth and, in addition, this expression reduced VIP receptor 2–Gαi interaction [236]. The data suggest that transmembrane domain 3–4 peptides are promising anticancer drugs.

### 2.3. Anticancer Peptides

#### 2.3.1. Angiotensin (1–7) Fragment

Angiotensin (1–7) is a biologically active peptide fragment of the renin–angiotensin system [237]. Angiotensin (1–7) has been shown in several studies to inhibit tumor cell proliferation [238]. It may reduce the growth of various cancer cell types, including lung, breast, prostate, and colorectal cancers [238]. Angiotensin (1–7) can inhibit angiogenesis via downregulation of pro-angiogenic factors like VEGF [239] and it can reduce cancer cell migration and invasion, potentially limiting metastatic spread [239].

Angiotensin (1–7) fragment, the product of angiotensin-converting enzyme 2, and the non-peptide MAS-R agonist AVE0991 (mimics the effects of angiotensin (1–7)) decreased the migration/invasion of BC cells [28]. Angiotensin-converting enzyme 2 expression was low in BC samples, and a high expression has been related with a high response to chemotherapy and a low response to endocrine therapies [240]. Angiotensin (1–7) sensitized BC cells to chemotherapy, and a high level of angiotensin (1–7) in plasma has been linked with an improved response to chemotherapy [240].

#### 2.3.2. Ghrelin

Ghrelin is a peptide hormone primarily produced in the stomach, known for its role in stimulating appetite and regulating energy balance [241]. Beyond metabolism, ghrelin also influences cell proliferation, survival, and inflammation, which are important processes in cancer biology [241]. This gastric-derived peptide blocked the proliferation of BC cells (MDA-MB-231, T47D, MCF-7) [242]. However, another study demonstrated that ghrelin increased BC cell proliferation (MDA-MB-231, MDA-MB-435) and that a preproghrelin isoform was highly expressed in the MDA-MB-435 metastatic BC cell line in comparison with the expression observed in the benign MCF-10A breast epithelial cell line [243]. It has been reported in men that the expression of ghrelin is correlated to BC-specific survival and men with tumors expressing ghrelin showed a lower risk for BC death that those not having such expression [244]. Finally, the ghrelin gene has been associated with BC-specific mortality in women showing a low native American ancestry [245].

#### 2.3.3. Peptide YY

Peptide YY is a 36-amino-acid peptide hormone primarily secreted by the L-cells of the ileum and colon after meals [246]. Some cancers, especially neuroendocrine tumors of the gastrointestinal tract, may express Peptide YY or have altered Peptide YY levels [188]. Peptide YY or its receptors might be overexpressed or dysregulated in certain cancer types, though this is tumor-specific [188]. Peptide YY and its fragments inhibited BC cell growth, migration and invasion [188]. Peptide YY blocked the growth of MCF-7 BC cells in vivo and decreased the level of cAMP in these cells [247]. The co-administration of vitamin E and peptide YY showed a higher anticancer effect than the administration of peptide YY alone [248].

### 2.4. Other Bioactive and Non-Bioactive Peptides and Breast Cancer

#### 2.4.1. ASRPS

ASRPS refers to a small regulatory peptide—a short chain of amino acids—that plays a role in modulating cellular processes [249]. Small regulatory peptides often act as signaling molecules by binding to specific receptors on cells, influencing pathways related to growth, differentiation, apoptosis, and other key cellular functions [249]. ASRPS can influence cancer cell behavior by regulating signaling pathways that control cell proliferation, migration, and survival. Depending on the context, ASRPS may act as an oncogenic factor promoting tumor growth or, alternatively, as a tumor suppressor inhibiting cancer progression. ASRPS may also modulate the tumor microenvironment, affecting angiogenesis, immune cell infiltration, or extracellular matrix remodeling. Due to its regulatory role, ASRPS or its receptors could serve as potential biomarkers or targets for anticancer therapies, either by blocking its action in tumors where it promotes growth or by mimicking/enhancing it where it suppresses tumors.

The downregulation of ASRPS (a STAT3 small regulatory peptide) in triple-negative BC cells has been associated with poor overall survival; this peptide downregulated STAT3 phosphorylation and reduced angiogenesis in an experimental animal model of BC; ASRPS downregulation favored tumor growth and the peptide acted as an anticancer peptide in in vivo experiments [250]. ASRP has conflicting roles in cancer; further investigations are necessary to define its role in cancer pathogenesis, pending which analogs/antagonists could be used as appropriate for BC treatment.

#### 2.4.2. Carnosine

Carnosine is a naturally occurring dipeptide composed of beta-alanine and histidine. It is found in high concentrations in muscle and brain tissues [251]. Known for its antioxidant, antiglycation, and metal-chelating properties [251], carnosine can reduce oxidative stress and inhibit advanced glycation end products, which are linked to cancer progression. By limiting oxidative DNA damage and protein modifications, carnosine may help protect cells from transformation [251,252]. Carnosine can inhibit the proliferation of certain cancer cell lines, such as glioblastoma, colorectal, and BC cells. It may act by interfering with energy metabolism, particularly by reducing glycolysis (the Warburg effect), which many cancer cells rely on [253]. Carnosine promotes cancer cell death and halts cell cycle progression. Due to its low toxicity and antioxidant properties, carnosine has been proposed as a supplement to enhance the effectiveness of chemotherapy and radiotherapy, possibly reducing side effects [254].

Carnosine exerts an anticancer effect against BC cells, blocking their proliferation [253,255]. This dipeptide decreased the activity of cytochrome C oxidase and the levels of VEGF, ATP and cyclin D1 in vitro and reduced BC growth in vivo [253]. Importantly, carnosine was not toxic to healthy cells [253]. Carnosine exerted an antiproliferative action against MDA-MB-231/EMT-6 BC cells; however, a modest increase in proliferation was found when MCF-7 BC cells were treated with the dipeptide [256]. Moreover, carnosine did not exert either toxic or proliferative effects on luminal cell lines, and the expression of angiotensin-converting enzyme 2 was reduced after the administration of carnosine [256]. The effects of different concentrations of L-carnosine from Karnozin EXTRA supplement on MCF-7 BC cells have been reported [257]. L-carnosine reduced cell number and viability, changed morphological characteristics, increased CYP2E1 expression, and reduced the activity of NADH-ubiquinone oxidoreductase, succinate dehydrogenase and cytochrome C oxidase [257].

The anticancer effect of carnosine-loaded niosomes (Car-Nio) on BC cells has been reported [258]. MCF-7 BC cells were arrested at the G2/M phase and MDA-MB-231 BC cells at the S phase, and the expressions of caspase 3/9, Bcl2-associated protein and protein 53 were upregulated after treatment with Car-Nio, whereas those of microRNA-183, poly (ADP-ribose) polymerase and B-cell lymphoma 2 were downregulated [258]. Another strategy to deliver carnosine to fight BC is the use of pegylated liquisomes, a combined passive targeting nanoplatform of L-carnosine [259]. Compared to the treatment with a carnosine solution, the use of pegylated liquisomes showed a higher anticancer activity (decreased tumor growth and cyclin D1 and VEGF levels; increased caspase 3 level) [259]. L-carnosine-coated magnetic nanoparticles have been developed and tested using MCF-7 BC cells [255]. This strategy decreased tumor size and the levels of cyclin D1 and VEGF. Previous data show some of the strategies that are currently being developed to deliver the anticancer dipeptide carnosine.

#### 2.4.3. Cocaine- and Amphetamine-Regulated Transcript

Cocaine- and amphetamine-regulated transcript (CART) is a peptide originally identified for its role in the central nervous system, regulating appetite, stress, and reward pathways [260]. Recently, research has explored its involvement in cancer biology, revealing some interesting and potentially significant findings: CART, initially studied in neuroscience, is gaining attention in oncology for its modulatory effects on cancer cell proliferation, survival, and metastasis [261,262].

It represents a promising target for future cancer diagnostics and therapeutics, although research is still preliminary [262]. Studies suggest CART can influence cancer cell proliferation, apoptosis, migration and invasion [262]. CART exerts its tumorigenic effects mainly via activation of G protein-coupled receptors, leading to intracellular signaling cascades such as cAMP/PKA pathway, MAPK/ERK pathway, and PI3K/Akt pathway [263].

Primary and metastatic BCs express CART, and this expression is an independent poor prognostic factor in lymph node-negative and estrogen receptor-positive BC [262,264]. CART expression in estrogen receptor-positive BC cells protected against cell death mediated by tamoxifen [264], and CART has been suggested as a biomarker in BC [262].

#### 2.4.4. Dynorphin

Dynorphins are endogenous opioid peptides that primarily bind to kappa opioid receptors. They are involved in modulating pain, stress, and emotional responses [265]. Dynorphin’s role in cancer is complex and not fully defined [266]. It may have both pro- and antitumor effects depending on tumor type, receptor expression, and microenvironment [267]. Fragments of dynorphin A (1–8, 1–17) have been reported in a carcinosarcoma (Walker 256 tumor) originating from a rat mammary gland [267]. Moreover, kappa opioid receptor 1 mediated the migration of BC cells and it is overexpressed in these cells (MDA-MB-231, MDA-MB-435, MCF-7) [268]. In this study, kappa opioid receptor 1 knockdown blocked BC cell viability and migration, reduced protein and gene expression of vimentin, snail, N-cadherin and matrix metalloproteinase 2, increased the expression of E-cadherin, and favored the inactivation of the PI3K/Akt signaling pathway [268]. Akt inhibition decreased cell viability and favored BC cell death, whereas the activation of Akt reversed the kappa opioid receptor 1 knockdown-promoted BC cell viability and cell migration blockade [268].

#### 2.4.5. Galanin

Galanin is a peptide consisting of 29–30 amino acids and is widely expressed in the central and peripheral nervous systems, as well as in several non-neuronal tissues [269]. Its roles in cancer are complex and context-dependent, with evidence of both tumor-promoting and tumor-suppressing functions, depending on the receptor subtype expressed and the cancer type [4]. Pre-pro-galanin mRNA and galanin expressions have been reported in BC and an increase in the expression of preprogalanin mRNA was not observed with GALN gene (which encodes the preprogalanin protein) amplification [270,271]. Galanin has conflicting roles in cancer; further investigations are necessary.

#### 2.4.6. HMK (HER2 Affibody-Matrix Metalloproteinase 2-Sensitive Cleavage Sequence-KLA (Kytoplasmic Lipid-Associated))

A genetically engineered fusion protein, named HMK, composed of pro-apoptotic peptide R8-KLA and HER2 affibody, promoted apoptotic mechanisms in SKBR3 BC cells through the activation of exogenous and endogenous apoptotic pathways [272]. HMK fusion proteins are promising targeted cancer therapies designed to minimize off-target toxicity, maximize tumor specificity, and trigger intrinsic apoptosis in tumor cells [272].

#### 2.4.7. KLA Peptide

The KLA peptide, often referred to as (KLAKLAK)2 or (KLAKLAK)2-amide, is a synthetic pro-apoptotic peptide that has shown promising anticancer properties, especially when conjugated with targeting moieties [273]. This peptide promoted apoptosis in tumor cells, and a chimera (smac-KLA) fusing a modified KLA peptide and an octa-peptide from the N-terminus of mature Smac protein (second mitochondrial-derived activator of caspases) exerted an anticancer synergic action by inducing apoptosis in BC cells (MDA-MB-231, MCF-7) and, in addition, favored an anti-inhibitor of apoptosis protein activity (drug resistance is caused by an upregulation of this inhibitor) [274]. This is important because the inhibitor of apoptosis protein blocked apoptosis via caspase inhibition, which was antagonized by Smac and, importantly, Smac-based peptides alone did not exert an effective anticancer action [274]. Monomeric and dimeric chimeras were tested in this study, and it was reported that the dimerization increased the anticancer effect 2–4-fold [274].

Several strategies have been developed to deliver (BRBP1-TAT-KLA)/enhance the activity (HPRP-A1) of the pro-apoptotic KLA peptide (disrupts the mitochondrial membrane) to exert an anti-BC effect (apoptosis in MCF-7 BC cells, reduction in tumor weight/volume, metastasis decrease), and importantly, normal tissues were not affected [275,276,277]. The hybrid peptide AFP-KLA promoted apoptosis in MCF-7 BC cells by disrupting the mitochondrial membrane [278]. In addition, this peptide also decreased tumor growth in in vivo experiments [278].

#### 2.4.8. LINC00511-133aa

LINC00511-133aa is a micropeptide encoded by the long non-coding RNA (lncRNA) LINC00511, and recent research has begun to uncover its role in cancer biology, especially in tumor progression and immune evasion [279]. This peptide is expressed in various cancers and is emerging as an oncogenic factor [279]. LINC00511-133aa was found to cause immune evasion in triple-negative BC by enhancing PD-L1 expression; facilitating interaction with CMTM6; stabilizing PD-L1 on the tumor cell surface; leading to reduced T cell cytotoxicity; enhancing tumor growth, tumor proliferation, and metastasis; and causing cancer stemness and drug resistance [279]. LINC00511-133aa favored stemness and invasiveness of BC cells (MCF-7, MDA-MB-231) via the activation of the Wnt/β-catenin signaling pathway [280].

#### 2.4.9. Melittin

Melittin, the main component of bee venom (from *Apis mellifera*), has been extensively studied for its anticancer properties in preclinical models. Anticancer mechanisms of melittin include apoptosis induction, anti-angiogenic effects, and suppression of oncogenic pathways like PI3K/Akt/mTOR (mammalian target of rapamycin), NF-κB and MAPK [281]. Melittin, through the downregulation of NFκB gene expression, reduced the levels of HIF-1α protein/mRNA; the inhibition of HIF-1α also promoted the downregulation of the expressions of lactate dehydrogenase A and VEGFA [282]. Moreover, melittin blocked the growth of MDA-MB-231 BC cells by activating apoptotic pathways and upregulating the expressions of Bax and tumor necrosis factor A [282].

Table 1 summarizes the main effects exerted by oncogenic and anticancer peptides in BC development.

## 3. Perspectives and Future Research

In 2040, 28.4 million individuals are expected to have cancer [1]; this is a major global health challenge, and therefore, new specific anticancer strategies must be sought and developed. As indicated in the previous section, peptidergic systems are involved in BC development, since bioactive peptides facilitate or counteract their progression. Peptide receptors are involved in cell communication and transformation, proliferation, apoptosis, invasion, migration and survival, and when these receptors do not adequately achieve cell functions, tumors can appear. The overexpression of peptide receptors in tumor cells opens an opportunity to allow for the selective destruction of these cells using peptide receptor agonists or antagonists. Thus, peptide receptors play an essential role in BC research and allow anticancer tailored pharmacological approaches to inhibit those signaling pathways which promote cell disturbances [3]. For example, neurokinin receptor 2 mediates BC cell proliferation, but this receptor is not involved in the proliferation of normal cells [177]. In sum, a full understanding of the roles played by the peptidergic systems in BC will serve to improve diagnosis and treatment.

The peptidergic system (e.g., gastrin-releasing peptide, endothelin, kisspeptin, neuromedin U, neuropeptide Y, neurotensin, oxytocin, substance P, angiotensin II) has been associated with BC progression [9,38,117,120,139,150,153,157,164,183,188,194,216,219,221,223,227,271]. Thus, the overexpression of the peptidergic system has been associated in BC with tumor size [120], the appearance of metastasis [221], an enhanced invasive/metastasis capacity of tumor cells [139,164], to lymph node positive grade [117], to lymph node metastasis and poor survival [153], an enhanced lymph node metastatic spread [227], higher aggressiveness, worse sensitivity to chemotherapeutic drugs and increased relapse risk [216,219], the risk of developing chemotherapy-induced cardiotoxicity [157], an improved response to chemotherapy [240], perineural invasion and advanced stages [188,194,195], a higher risk for BC recurrence [150], poor outcomes [183], shorter survival times [164], and Ki-67 and higher pT stage [223]. However, the overexpression of the peptidergic systems does not always mean a harmful effect. Patients with a high expression of gonadotropin-releasing hormone receptors exhibited better disease-free survival than those showing a lower expression [96], and healthy women showed an elevated level of β-endorphin, whereas in women suffering from BC a lower level of β-endorphin was reported [68]. On the contrary, less elevated kisspeptin expression has been associated with a negative prognostic factor for overall survival, axillary lymph node status, metastatic propensity, advancing tumor stage, and advanced grade [121]. In addition, low AM expression has been associated with an augmented risk of metastasis and recurrence and poor prognosis [17]. These are some examples confirming the complexity of the mechanisms involved and the participation of the peptidergic systems in the development of BC, and this means that the peptidergic systems are promising therapeutic targets to fight BC.

As indicated above, there are peptides that exert an oncogenic action while others exert an antitumor action. Several peptides exert a dual action, playing a role in both oncogenic and anticancer effects in BC. Many in vitro and in vivo experiments blocking the action of oncogenic peptides have been performed with excellent anti-BC effects. Thus, anti-AM2 antibodies [146], the silencing of endothelin receptor B [153], GLP 1 receptor antagonists (exendin (9–39)) [86], kinin B1 and B2 receptor antagonists [38], endothelin receptor A/B antagonists (bosentan, macitentan) [151,154], neurokinin receptor 2 antagonists (neurokinin receptor 2 mediates the proliferation of BC cells but it is not involved in the proliferation of normal cells) [177], neuromedin B receptor antagonists (PD168,368) [180,181], neuropeptide Y receptor antagonists (CGP71,683A) [188], neurotensin receptor antagonists (SR48,692)/neurotensin receptor 1 silencing [218,219,220] and neurokinin receptor 1 antagonists (aprepitant) [177,228,229] blocked BC cell proliferation, migration, invasion, metastasis and adherence, and angiogenesis, favored BC cell cycle arrest, augmented apoptosis in BC cells, decreased the EMT of BC cells, reduced tumor growth and lung metastasis of BC cells, and sensitized experimental BC brain metastases to paclitaxel [38,86,112,146,151,153,156,177,180,181,188,218,219,220,228,229]. Therefore, many experiments have confirmed the use of peptide receptor antagonists as antitumor agents against BC. Furthermore, the use of anticancer peptides to treat BC is also possible, and many experiments have demonstrated this anti-BC strategy. Thus, AM [17], angiotensin (1–7) [28,240], kinin agonists (FR190,997) [39,283], CRF [52,55,60,61], β-endorphin [66,67], ghrelin [242], GLP 1 receptor agonists (semaglutide, liraglutide, exendin 4) [78,79,80,84], gonadotropin-releasing hormone analogs (goserelin (Zoladex)) [12,96], kisspeptin [124,126,127], peptide YY [247], carnosine [253,284] and melittin [282] blocked BC cell proliferation, migration, invasion and metastasis, favored apoptosis, decreased the motility of BC cells, tumor volume and colony formation, increased the expression of peptide receptors in BC cells, promoted G0/G1 phase arrest, decreased DNA synthesis, counteracted angiogenesis, sensitized BC cells to chemotherapy, increased the action of chemotherapy, increased acquired antitumor immunity, favored immune-mediated antitumor defenses inhibiting BC development, blocked the EMT in BC cells, reversed the Warburg metabolic switch in BC cells, impaired glycolysis, downregulated Twist1/Snail1 and N-cadherin, and upregulated E-cadherin [12,17,28,39,52,55,60,61,66,67,78,79,80,84,96,124,126,127,240,242,247,253,282,283,284]. In sum, previous data confirm that peptide receptor (AM2, GLP 1, kinin, endothelin, neurokinin, neuromedin B, neuropeptide Y, neurotensin) antagonists and peptides/peptide receptor agonists (AM, angiotensin (1–7), kinin, CRF, β-endorphin, ghrelin, GLP 1, gonadotropin-releasing hormone analogs, kisspeptin, peptide YY, carnosine, melittin) could be used as antitumor agents alone or in combination therapy because they exert numerous and varied actions against the development of BC. Peptides show a short half-life and poor bioavailability but they are safe and have a high solubility; currently, there are many strategies to counteract the drawbacks of peptide administration and to ameliorate the stability and delivery of peptides such as cell-penetrating peptides, cell-targeting peptides, peptide cyclization, peptide-loaded nanoparticles, peptide conjugation to polymers and amino acid sequence manipulation [11,285].

Knowing all which was previously detailed above, the question is how to establish a specific anti-BC strategy using these agonists or antagonists alone or in combination therapy. The answer is associated with the number of peptide receptors expressed in BC cells since, compared to those expressed by normal cells, tumor cells overexpress these receptors in general [7,8,9]. Thus, this overexpression can be used to attack BC cells with peptide receptor antagonists (favoring apoptosis in BC cells) when BC cell proliferation, migration and invasion are promoted by oncogenic peptides, with anticancer peptides/peptide analogs or with peptide receptor agonists/antagonists carrying antitumor cargo into BC cells. The FDA has approved the use of peptide analogs (e.g., gonadotropin-releasing hormone, somatostatin) for the diagnosis and treatment of some tumors and, in fact, the gonadotropin-releasing hormone analog named goserelin (Zoladex) has been approved for the palliative treatment of advanced BC [11,12,286]. This is an example of the therapeutic potential that the peptidergic systems have in the treatment of BC and, in addition, current knowledge about peptide receptor antagonists supports that these antagonists could be approved soon for clinical use as antitumor drugs. The problem is how to act when the same peptide promotes an oncogenic action and acts as an antitumor agent (e.g., CRF favored MCF-7 BC cell motility and invasiveness [54] but CRF also blocked the migration of these cells [60]). This is an example of the importance of knowing the expression of the different types of peptide receptors expressed by the same BC cell (e.g., MCF-7); if the expression of oncogenic receptors (e.g., neurokinin 1) is demonstrated in addition to CRF receptors in MCF-7 cells, these cells could be treated with the neurokinin 1 antagonist aprepitant, which promotes apoptosis, and thus the use of CRF peptide receptor agonists or antagonists to treat MCF-7 cells is avoided due to the double effect (oncogenic–anticancer) exerted by CRF on MCF-7 cells. Furthermore, different types of cancer overexpress the same peptide receptor and this means that if, for example, this receptor mediates an oncogenic response, an anticancer treatment with the same apoptotic peptide receptor antagonist (e.g., aprepitant) could be applied. Moreover, peptide receptor overexpression serves a purpose in the imaging, diagnosis and treatment of BC using, for example, 68Ga/177Lu-labeled angiotensin II or applying strategies to deliver chemotherapeutic drugs [8,9,99,102,104,105,107,201,206,207,208,209,259]. These strategies successfully blocked BC cell proliferation, promoted apoptosis in BC cells, increased caspase 3 level, and decreased tumor growth and cyclin D1 and VEGF levels [102,103,104,105,259]. Importantly, the fusion of LHRH to its pore-forming domain (BinBc) blocked the proliferation of BC cells overexpressing LHRH receptors, but this compound did not affect human fibroblast cells [105]. This is an example of the specificity and safety of anti-BC strategies. Moreover, it has been reported that anticancer peptides from a random peptide library reduced the survival and proliferation of BC cells by activating intrinsic apoptotic pathways, but they did not affect fibroblasts or normal mammary epithelial cells [287].

According to current knowledge, the same peptide (e.g., AM, CRF, kisspeptin, methionine-enkephalin) exerts a dual action (oncogenic and anticancer) in BC. For example, CRF favored the motility and invasiveness of MCF-7 BC cells [54] but another experiment showed that CRF inhibited the migration of these cells [60]; carnosine exerted an antiproliferative action against MDA-MB-231/EMT-6 BC cells, but an increase in proliferation was found when MCF-7 BC cells were treated with the dipeptide [256]. Carnosine was not toxic to healthy cells [253]. KP-10 promoted the migration of MDA-MB-231 BC cells but not that of BT-20 BC cells [118]; kisspeptin exerted an antimetastatic action in some cancers (lung, colon) but in BC favored aggressiveness and aggravated BC prognosis [112], and methionine-enkephalin promoted the migration of MDA-MB 231 BC cells [71] but inhibited the proliferation of these triple-negative BC cells [72]. It is crucial to understand why this dual action occurs as well as to know the mechanisms that regulate it. The oncogenic and anticancer properties of peptides could be due to the specific characteristics of the cell or tumor type studied, to the peptide receptor type expressed, to the G protein activated (a peptide can bind to several G protein-coupled receptors activating several signaling pathways), to the specific signaling pathway involved, to the tumor microenvironment milieu, and to different experimental procedures. In this sense, it is known that bradykinin, via kinin B1 and B2 receptors, activated the migration/invasion of BC cells through the FAK/Src signaling pathways [38]; that exendin 4 counteracted BC cell growth by blocking nuclear factor κB (NF-κB) [76]; that kisspeptin blocked metastatic SKBE3 BC cell growth, migration and metastasis through the activation of eukaryotic translation initiation factor 2α kinase 2 (EIF2AK2) [124]; and that melittin blocked the growth of MDA-MB-231 BC cells by activating apoptotic pathways and upregulating the expressions of Bax and tumor necrosis factor A [282]. Therefore, previous signaling pathways and factors associated with the peptidergic systems are also potential therapeutic targets to fight BC. This is a crucial research line that merits development.

It is also crucial to understand how peptidergic systems are regulated in BC and how oncogenic and anticancer peptides interact with each other in this disease. In this sense, melatonin, via the expression of kisspeptin, blocked triple-negative BC metastasis [129]; stromal-derived factor-1 favored the invasion of BC cells, which was blocked with antibodies directed against this factor [130]; fibulin 3 regulated triple-negative BC metastasis [115], and estrogens augmented the mRNA encoding CRF receptor 2 and a splice variant encoding CRF receptor 1 (this variant increase reduced the cell response to CRF and prevented its repressive action on BC cell invasion) [56]. Moreover, it is crucial in BC cells to know the factors that control the synthesis/release of anticancer and oncogenic peptides, as well as to know how the gene expression of the different peptide family precursors is regulated. It is also important to conduct in-depth experiments studying the different peptide receptors that a single human BC cell line expresses and how the co-administration of oncogenic and anticancer peptides affects BC cell proliferation, migration and metastasis, as well as angiogenesis. It is worth knowing whether or not there is an anti-BC synergistic action when different peptide receptor antagonists favoring apoptosis are administered or when peptide receptor antagonists and anticancer peptides are co-administered. It is important to know the interactions between oncogenic and anticancer peptides in BC as well as to better understand the functional complexity of the mechanisms controlling the synthesis and release of peptides in BC development. Moreover, the combination therapy of peptide receptor antagonists or anti-BC peptides with radiotherapy, chemotherapy or immunotherapy must be studied in depth in BC; in this sense, it is crucial to study whether these antagonists and peptides reduce (or not) the side-effects mediated by cytostatic drugs. It has been demonstrated that the co-administration of anticancer peptides/GLP 1 inhibitors and other compounds is a useful strategy to fight BC. Thus, the co-administration of exendin 4 (GLP 1 blocker) and metformin (promotes apoptosis) counteracted BC progression [84], and the co-administration of vitamin E and peptide YY showed a higher anti-BC effect than the administration of peptide YY alone [248]. These are examples of the importance of studying and applying combined therapies to fight BC. Moreover, the co-administration of dasatinib and doxorubicin using a multifunctional protein–nanodiamond nanocomposite has been performed via LHRH receptors; this strategy enhanced synergistic cytotoxic actions against MDA-MB-231 triple-negative BC cells, decreased tumor growth, and increased survival rate in in vivo experiments [95]. Moreover, the co-administration of cisplatin (a chemotherapeutic drug) and aprepitant (a neurokinin receptor 1 antagonist) increased the anticancer effects against triple-negative BC cells compared with the anticancer effect promoted when current therapies were applied; in addition, this antagonist decreased the harmful actions favored by chemotherapy [13,14]. Furthermore, the FDA has approved the gonadotropin-releasing hormone analog named goserelin (Zoladex) for the palliative treatment of advanced BC [11,12,286].

In addition, there are other BC research lines that are worth being studied in-depth and developed: (1) the involvement of peptide receptors in the viability of BC cells (e.g., the silencing of endothelin receptor 1 promotes apoptosis in BC cells) [150] and the mechanisms regulating the overexpression of peptide receptors (e.g., neurokinin A increases the expression of neurokinin receptors 1 and 2; substance P increases the expression of neurokinin receptor 1) [178]; (2) the roles that peptide fragments play in the development of BC (e.g., angiotensin II did not affect the invasion of BC cells but the angiotensin (1–7) fragment decreased the migration and invasion of these cells) [28]; (3) the roles of peptides in favoring immune-mediated anti-BC defenses [66,67]; (4) to know how peptides change the tumor microenvironment [63]; (5) to study the involvement of the peptidergic systems in drug resistance [113,184,185,186]; (6) how peptides block the Warburg effect [128]; (7) contradictory findings, such as how CRF favors MCF-7 BC cell motility and invasiveness [54] but CRF also blocked the migration of these cells [60]; (8) to increase the knowledge on the actions carried out by ASRPS, HMK, KLA, LINC00511-133 aa, ghrelin, dynorphin, carnosine, and melittin in BC [250,272,274,280,282]; (9) to confirm and study in depth the use of the peptidergic systems as biomarkers (e.g., CART, neuropeptide Y receptor 1) [197,262]; (10) to confirm that somatostatin receptor 2 is a promising therapeutic molecular target in triple-negative BC [286]; (11) to elucidate the involvement of oxytocin in the beneficial effects of exercise training on BC [144]; (12) to investigate the endothelin receptor blocker atrasentan as an agent that improves cardiac functions and reduces cardiac remodeling in BC [159]; (13) to study in depth the antiproliferative actions against BC cells mediated by the kinin receptor B2 agonist FR190,997 [39]; (14) the use of single-nucleotide polymorphisms rs5370 in endothelin 1 to identify patients who are unlikely to gain any advantage from bevacizumab [160]; (15) to investigate the relationship between the rs5780218 polymorphism of the kisspeptin 1 gene and an increased risk of BC development [123]; (16) epigenetic mechanisms to know how they regulate the peptidergic systems; and (17) the use of machine learning models to diagnose BC using serum biomarkers (e.g., oxytocin) [288].

It should be noted that in addition to the numerous research lines previously mentioned, others are very promising for the diagnosis and treatment of breast cancer, such as transferrin receptors and radiolabeled mucin or bioactive peptides [9,168,289,290,291,292,293,294]. Thus, a high expression of transferrin receptors has been reported in primary and metastatic breast cancer and after neoadjuvant chemotherapy and hence transferrin conjugation to imaging or nanotherapeutic agents is a potential and promising therapeutic strategy to fight breast cancer [289]. In this study, systemic iron chelation with deferoxamine increased transferrin receptor uptake in breast cancer cells, and this means that this iron chelator could ameliorate the uptake of potential tracers or therapeutics that use transferrin as a ligand [289]. Moreover, the authors suggested that deferoxamine could upregulate receptor expression [289]. In this sense, a nanoconjugate carrying pH-responsive transferrin receptor-targeted hesperetin (a natural bioflavonoid with antitumor effects) promoted triple-negative breast cancer cell death via assemblage of pro-apoptotic proteins and oxidative attack [290]. This nanoconjugate increased the intracellular level of reactive oxygen species, dropped the potential of the mitochondrial membranes, and promoted apoptosis by arresting the breast cancer cell cycle at the G0/G1 phase [290]. The use of mucin-1-conjugated polyamidoamine-based nanoparticles for image-guided delivery of the targeted cancer drug gefitinib is also a promising strategy to treat breast cancer cells/tumors expressing the mucin-1 receptor with the assistance of nuclear medicine [291]. In this study, nanoparticles were radiolabeled with the image agent gallium-67, and a high cytotoxicity was observed in breast cancer cells in in vitro experiments; in addition, nuclear medicine imaging showed the accumulation of these nanoparticles in tumors in in vivo experiments [291]. Moreover, it is known that an overexpression of the cancer-associated antigen transformed mucin-1 occurs in triple-negative breast cancer cells [292]. The humanized TAB004 antibody, targeting the transformed mucin-1, showed a theranostic effect against triple-negative breast cancer cells and, in fact, ^225^Ac-DOTA-hTAB004 augmented survival and decreased tumor volume [292]. This is another promising theranostic strategy to fight breast cancer. Finally, bioactive radiopeptides are widely used in nuclear medicine for both diagnosis and treatment of breast cancer [9,168,293,294]. In this sense, the radiolabeled leuprolide peptide (a gonadotropin-releasing hormone analog) with therapeutic (^177^Lu) or diagnostic (^68^Ga) radionuclides is a promising therapeutic strategy to detect and treat breast cancer [294]; radiolabeled (^177^Lu/^68^Ga) angiotensin II peptide could also be used to diagnose and treat breast cancer [9]; radiolabeled (^44g^Sc/^68^Ga) bombesin antagonists, targeting gastrin-releasing peptide receptors, have been suggested as radiotheranostics to treat breast cancer [293], and the same receptors have been suggested as promising targets for the development of theranostic radioligands to treat luminal breast cancer with positive estrogen receptor expression; this is important because gastrin-releasing peptide receptors are also expressed in distant metastasis and lymph nodes [168].

## 4. Conclusions

**Figure 1:**
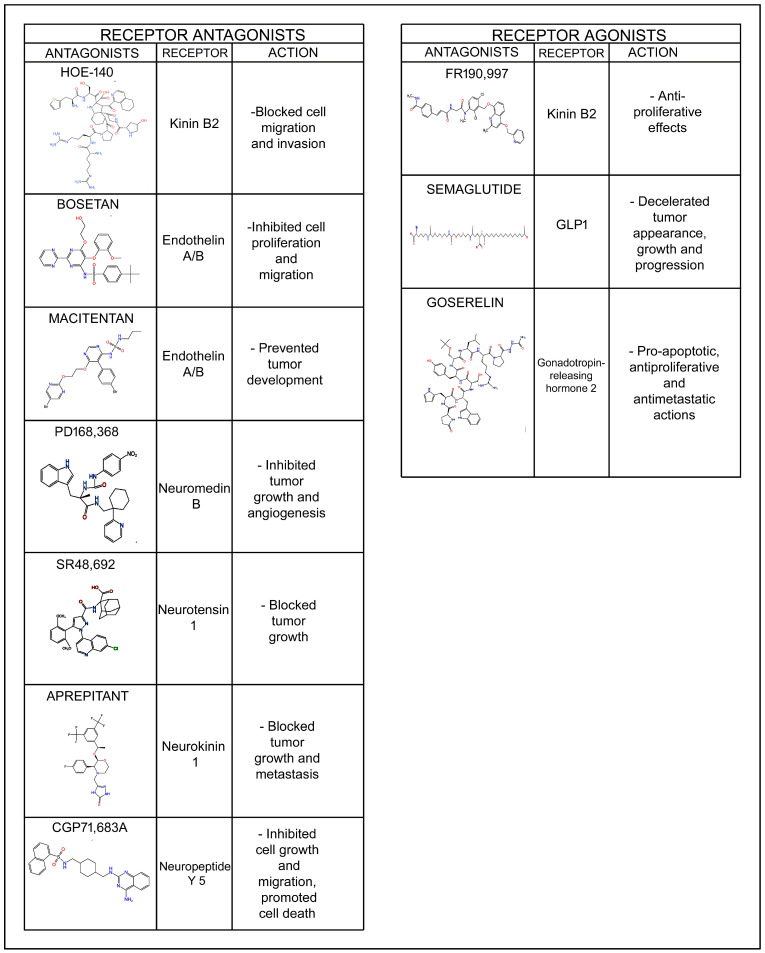
Chemical structures of peptide receptor antagonists/agonists exerting an anti-BC effect. The receptors involved and the anti-BC actions exerted are also indicated [38,39,84,92,93,94,151,154,180,193,220,227].

**Figure 2:**
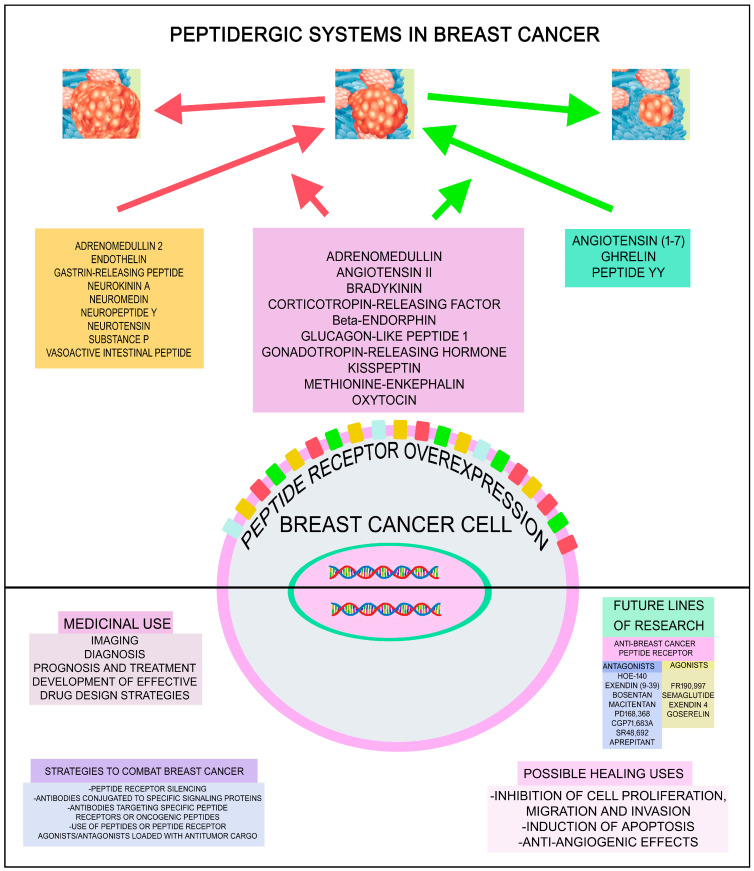
Anti-BC therapeutic strategies. Green arrows: anticancer peptides. Red arrows: oncogenic peptides. Future lines of research are also indicated.

The current known data on the involvement of the peptidergic systems in BC progression are overwhelming. The data reported in this review confirm the complexity of the involvement of the peptidergic systems in the development and treatment of BC because peptides can exert oncogenic action, antitumor action, or dual oncogenic and antitumor action. Peptidergic systems are useful tools for imaging, diagnosis, prognosis and treatment of BC, and these systems are key pieces in basic and clinical BC research by exploring new molecular mechanisms, signaling pathways, and adequate drug design strategies. Understanding how these peptidergic systems are regulated in BC in relation to the anticancer and/or oncogenic profile of the peptide is crucial and, in addition, understanding the potential side effects will be useful for combination therapies. Developing specific peptidergic anti-BC strategies will likely be based on the expression of peptide receptors in BC compared to normal tissues; this peptide/receptor pair may generate differential responses in different tumor types or individuals depending on the aggressiveness and expression within the disease, which warrants further exploration. Future studies must confirm that peptide receptor antagonists or peptide agonists do not interfere with the physiological mechanisms in healthy cells and, if confirmed as it seems, anti-BC strategies will be specific and safe. Thus, stable, durable, safe and specific anti-BC drugs must be developed against BC cells without touching healthy cells. It is crucial to examine peptide receptor structure and signaling to foster innovative pharmacological solutions to adapt, modulate, and mend peptide receptor dysfunctions [3]. Figure 1 shows promising anti-BC peptide receptor antagonists or agonists. Different types of cancer overexpress the same peptide receptor, and this means that if, for example, this receptor mediates an oncogenic response, an anticancer treatment with the same peptide receptor antagonist could be applied. Moreover, drugs for two or more molecular targets must be designed and, in addition, they must complement other anti-BC treatments (chemotherapy, radiotherapy, immunotherapy). Many anti-BC strategies are possible: peptide receptor silencing, antibodies conjugated to specific signaling proteins, antibodies against specific peptide receptors, antibodies against oncogenic peptides, peptides/peptide receptor agonists with antitumor cargo and peptide receptor antagonists. Drug cocktails (e.g., peptide receptor antagonists and anti-BC peptides) would be beneficial for the treatment of BC alone or in combination therapy with radiotherapy, chemotherapy and immunotherapy. Peptidergic systems have great anti-BC clinical potential that must be exploited and developed. Taken together with the available current data, the possibility of transversal research regarding BC and the peptidergic systems is enormous and quite promising to treat BC. In summary, a full understanding of the roles played by the peptidergic systems in BC will serve to improve diagnosis and treatment. Figure 2 summarizes the anti-BC therapeutic strategies mediated by the peptidergic systems.

## Figures and Tables

**Table 1:** Peptides favoring and/or counteracting BC development.

Oncogenic and Anticancer Peptides		
Peptides	Actions	
	Oncogenic	Anticancer
Adrenomedullin	- Accelerated bone metastasis [18] - Fibroblasts release AM, promoting angiogenesis and tumor growth [19]	- Blocked cell invasion and metastasis [17]
Angiotensin II	- Angiotensin II receptor 1 overexpression favored angiogenesis and tumor growth [9]	- Decreased cell motility [28]
Bradykinin	- Facilitated migration and invasion; both effects blocked with kinin B1 and B2 receptor antagonists (Des-[Arg9]-Leu8-bradykinin, HOE-140) [38] - Bradykinin analogs promoted cell proliferation and metalloproteinase 2/9 release, favoring invasion and metastasis [41,42] - BC cell stimulation with kinin B1 receptor agonists increased peptidase KLK6/KLK11 levels (favoring invasiveness and proliferation) and decreased KLK10 (protease related to growth suppression) level [41] - Kinin receptor B1 antagonists cooperated with chemotherapeutic drugs (paclitaxel, doxorubicin) to favor the death of triple-negative BC cells [40]	- Kinin receptor B2 agonists (FR190,997) exerted antiproliferative effects [39]
Corticotropin-releasing factor	- CRF receptor 2 mediated cell migration [51] - Mediated cell proliferation, migration, invasion, and metastasis and regulated the immune response [57] - Favored cell motility and invasiveness, blocked apoptosis, augmented FAK phosphorylation and actin polymerization and promoted the synthesis of prostaglandins, favoring metastasis [54]	- Inhibited cell growth [55] and migration [60] - Tumor suppressor [60] - Decreased tumor volume without affecting angiogenesis and increased chemotherapy action [61] - CRF and urocortin 2 promoted apoptosis [52]
Endorphin	- Activated mitogenic and survival pathways [65]	- Blocked BC development by favoring immune-mediated antitumor defenses [66,67] - Blocked sympathetic neuronal action, increasing the synthesis of anti-inflammatory cytokines and the activities of macrophages and natural killer cells [63] - Beta-endorphin-transplanted animals showed a decrease in mammary tumor incidence, malignancy rate, growth and metastasis, epithelial-to-mesenchymal transition and inflammatory processes [69] - Beta-endorphin neuron transplants augmented macrophage and natural killer cell activities, decreased plasma levels of inflammatory cytokines, and augmented anti-inflammatory cytokine plasma levels [69]
Enkephalin	- Methionine-enkephalin promoted cell migration [71] - Low fasting pro-enkephalin plasma level in postmenopausal middle-aged women related to augmented BC risk development [74]	- Methionine-enkephalin inhibited cell proliferation [72]
Glucagon-like peptide 1	- Liraglutide favored cell growth and accelerated BC; exendin (9–39) inhibited these effects [86,87]	- GLP 1 receptor agonists blocked tumor cell growth, promoted apoptosis and G0/G1 phase arrest, decreased colony formation and controlled angiogenesis [77,80] - Semaglutide decelerated tumor appearance and growth and increased acquired anticancer immunity and tumor infiltration [78] - GLP 1 analogs impaired glycolysis and blocked cell proliferation [79] - Exendin 4 decreased cell proliferation, DNA synthesis and tumor size [76,84] - Exendin 4 promoted apoptosis [85]
Gonadotropin-releasing hormone/luteinizing hormone-releasing hormone	- Leuprorelin (gonadotropin-releasing hormone receptor agonist) favored tumor progression and controlled gene expression associated with tumor progression [95]	- Goserelin (Zoladex) approved by the FDA for the palliative treatment of advanced BC [12] - Gonadotropin-releasing hormone receptor 2 analogs exerted pro-apoptotic, antiproliferative and antimetastatic actions [92,93,94] - Gonadotropin-releasing hormone receptor activation blocked cell proliferation and metastasis, promoted apoptosis, and increased the protein expression of gonadotropin-releasing hormone receptor in triple-negative BC cells [96] - Co-administration of Src/FAK inhibitors and gonadotropin-releasing hormone receptor antagonists (degarelix) counteracted BC growth and metastasis and augmented survival [95] - The immunotoxin (gonadotropin-releasing hormone-DNA fragmentation factor 40) promoted apoptosis and blocked cell invasive capacity [99] - Conjugated drugs (LHRH-conjugated paclitaxel; LHRH-conjugated prodigiosin) showed a higher anticancer effect (growth inhibition) against triple-negative BC cells than unconjugated drugs [102,103] - Pt-Mal-LHRH decreased triple-negative BC tumor growth [104] - BinBc blocked cell proliferation, but human fibroblasts were not affected [105] - BinBc promoted apoptosis [105]
Kisspeptin	- Promoted aggressiveness and aggravated prognosis [112] - Kisspeptin 1 receptor mediated tumor growth, cell invasion, and metastasis and favored drug resistance [113,116] - Favored invadopodia formation, cell invasion and metastasis [110] - Favored tumor growth and metastasis [116] - KP-10 promoted invasion and migration [114,117] - Less elevated kisspeptin expression associated with axillary lymph node status, negative prognostic factor for overall survival, advancing tumor stage and metastatic propensity [121] - Correlation between kisspeptin 1 receptor mRNA expression and tumor size and lymph node metastasis [120] - High level of kisspeptin 1 associated with lymph node-positive grade [117] - The rs5780218 polymorphism related to increased BC risk development [123]	- Blocked cell proliferation, migration and metastasis [124,125] - Counteracted angiogenesis of BC brain metastasis [126] - KP-10 blocked motility and migration, promoted apoptosis, inhibited tumor growth, blocked intratumoral blood microvessel formation, and improved survival rate [127] - KP-10 inhibited Warburg effect and promoted mitochondrial injury [128]
Oxytocin	- Oxytocin receptor overexpression associated with mammary hyperplasia and tumorigenesis [137] - High oxytocin receptor expression: increased cell migration and decreased survival [139] - High expression of oxytocin receptors associated with an enhanced metastasis capacity [139] - Oxytocin expression higher in BC subjects than in healthy individuals [138]	- Antiproliferative action [136]
**Oncogenic Peptides**		
Peptides	Actions	
Adrenomedullin 2	- Favored cell growth, migration, invasion and metastasis which were blocked with anti-AM2 antibodies [146] - Favored BC cell invasion and metastasis by increasing protein translation/ribosome biogenesis [146] - AM2 level correlated with Ki-67 expression and lymph node metastasis [146]	
Endothelin	- Increased invasiveness [149] - Favored Akt activation. Endothelin receptor 1 silencing promoted apoptosis [164] - Endothelin receptor B silencing reduced cell proliferation, migration and invasion, increased apoptosis and retarded the growth of implanted tumors [153] - Bosentan inhibited cell proliferation and migration mediated by endothelin 1 [151] - Macitentan prevented tumor development [154] and sensitized experimental BC brain metastases to paclitaxel [105] - Co-administration of macitentan and paclitaxel decreased tumor cell proliferation, increased overall survival and promoted apoptosis [105] - Endothelin 1-enriched tumor phenotype related to higher risk for BC recurrence [164] - Endothelin receptor B expression related to poor survival and lymph node metastasis [153] - The single-nucleotide polymorphisms rs5370 in endothelin 1 identify patients who are unlikely to gain any advantage from bevacizumab [160]	
Gastrin-releasing peptide	- Higher expression, shorter survival times [164] - Overexpression related to enhanced cell invasive capacity [164] - A knockdown of gastrin-releasing peptide reduced cell invasive capacity [164] - Gastrin-releasing receptors: potential theranostic target [7,165,166,167,168,169,170,171,172]	
Neurokinin A	- Promoted cell proliferation. Neurokinin receptor 1 and 2 overexpression in metastatic BC cells compared to non-metastatic ones [175,176] - Neurokinin receptor 2 mediated BC cell proliferation but not involved in normal cell proliferation [116] - Neurokinin receptor 2 antagonists inhibited cell proliferation [116] - Promoted migration and invasion and augmented aggressiveness [123] - Increased the expression of neurokinin receptors 1 and 2 in metastatic BC cells and favored the release of a bradykinin precursor that promotes tumorigenic effects [123]	
Neuromedin	- Neuromedin B: growth and pro-angiogenic factor [181] - Neuromedin U promoted the progression of cells expressing neuromedin U receptor 2 [115] - PD168,368 inhibited tumor growth and angiogenesis, promoted apoptosis and cell cycle arrest, blocked migration, invasion and metastasis and decreased the epithelial–mesenchymal transition [180,181] - Neuromedin U expression upregulated in BC tissue when compared to healthy breast tissue; this expression associated with poor outcome in breast tumors showing a strong neuromedin U receptor 2 expression [115] - Neuromedin U overexpression in HER2-positive BC cells augmented glycolysis [184] - Neuromedin U overexpression in drug-sensitive cells promoted resistance to HER-targeting drugs [126] - Neuromedin U in HER2-overexpressing BC cells increased resistance to anticancer immune responses [117] - Neuromedin U: a biomarker to predict and overcome resistance to HER-tyrosine kinase inhibitors and a useful tool to improve HER-targeted drug efficacy [126]	
Neuropeptide Y	- Promoted cell proliferation, migration, invasion, metastasis and angiogenesis [129] - Neuropeptide Y receptor antagonists inhibited all previous effects and favored tumor cell death [129] - CGP71,683A inhibited BC cell growth and migration and promoted the death of BC cells expressing neuropeptide Y receptor 5 [128] - Neuropeptide Y receptor 1/5 mRNA levels were augmented by hypoxia-inducible factors, promoting cell proliferation, migration and invasion [122] - Neuropeptide Y receptor 1 high expression associated with advanced stages, perineural invasion and lymph node metastasis [188,194,195] - High serum neuropeptide Y receptor 1 level positively correlated with clinical stage and lymph node metastasis; BC patients expressing neuropeptide Y receptor 1 had a shorter survival [194] - Tamoxifen promoted the loss of neuropeptide Y receptor 1 in BC cells [127] - Neuropeptide Y receptor 1 expression: a biomarker to predict better survival and endocrine sensitivity in estrogen receptor-positive BC subjects [130] - Neuropeptide Y analogs as specific BC-targeting agents [200] - Neuropeptide Y receptor overexpression in BC cells allows the use of designed compounds for breast tumor imaging and treatment [206,207,208,209]	
Neurotensin	- Promoted cell proliferation, migration, invasion, metastasis and an anti-apoptotic action. Neurotensin receptor antagonists/neurotensin receptor 1 silencing inhibited all previous actions [218,219,220] - Neurotensin receptor 1 antagonists blocked cell adherence, migration and invasion [216] - SR48,692 or the silencing of the neurotensin receptor 1 blocked tumor growth [218] - Favored HER2/3 and EGFR overexpression [216] - Plasma pro-neurotensin associated with BC development [217] - Neurotensinergic system overexpression correlated with higher aggressiveness, worse sensitivity to chemotherapeutic drugs, tumor size, poor prognosis and increased relapse risk [216,219] - Cytoplasmic neurotensin receptor 1 overexpression associated with higher pT stage and higher tumor grade; nuclear location correlated with lower pT stage, low Elston and Ellis grade and estrogen receptor positivity [223]	
Substance P	- Favored cell migration and invasion and increased aggressiveness [123] - The release of substance P from sensory nerve cells favored breast tumor growth, invasion and metastasis and aprepitant blocked BC growth and metastasis [217] - Increased neurokinin receptor 1 expression in metastatic BC cells but not neurokinin receptor 2 expression [123] - Neurokinin receptor 1, overexpressed in BC cells, mediated the viability of these cells; substance P promoted BC cell proliferation; neurokinin receptor 1 antagonists blocked mitogenesis and promoted apoptosis [222] - Neurokinin receptor 1 antagonists, including aprepitant, exerted antiproliferative, antimetastatic and apoptotic effects against triple-negative BC cells and decreased tumor volume [229] - Aprepitant is a promising candidate for BC treatment [222]	
Vasoactive Intestinal Peptide	- VIP receptor 2 involved in cell proliferation and migration [86] - BC cells expressing transmembrane domains 3–4 blocked lymph node metastasis and tumor growth [86]	
**Anticancer Peptides**		
Peptides	Actions	
Angiotensin (1–7)	- Decreased cell migration and invasion [28] and sensitized BC cells to chemotherapy [88] - High plasma level linked with an improved response to chemotherapy [88]	
Ghrelin	- Blocked cell proliferation [242]	
Peptide YY	- Blocked cell growth, migration and invasion [129] - Decreased cAMP level [91] - Co-administration of vitamin E and peptide YY: higher anticancer effect than the administration of peptide YY alone [89]	
**Other Peptides**		
Peptides	Actions	
	Oncogenic	Anticancer
ASRPS	- ASRPS downregulation favored tumor growth and associated with poor overall survival [250]	- Reduced angiogenesis and acted as an anticancer peptide [250]
Carnosine	- Increased MCF-7 BC cell proliferation [256]	- Antiproliferative action against MDA-MB-231/EMT-6 BC cells [256] - Not toxic to healthy cells [253] - L-carnosine reduced cell number and viability, changed morphological characteristics and increased CYP2E1 expression [257] - L-carnosine-coated magnetic nanoparticles decreased tumor size [255]
HMK		- Promoted apoptosis [272]
KLA peptide		- Promoted apoptosis as well as smac-KLA, reduced tumor volume and metastasis; normal tissues were not affected [274,275,276,277] - Favored an anti-inhibitor of apoptosis protein activity (drug resistance caused by an upregulation of this inhibitor) [274] - AFP-KLA promoted apoptosis and decreased tumor growth [278]
LINC00511-133aa	Favored stemness and invasiveness [280]	
Melittin		- Blocked cell growth by activating apoptotic pathways [282]

## Data Availability

Not applicable.
